# Involvement of Thyroid Hormones in Brain Development and Cancer

**DOI:** 10.3390/cancers13112693

**Published:** 2021-05-30

**Authors:** Gabriella Schiera, Carlo Maria Di Liegro, Italia Di Liegro

**Affiliations:** 1 Department of Biological, Chemical and Pharmaceutical Sciences and Technologies (Dipartimento di Scienze e Tecnologie Biologiche, Chimiche e Farmaceutiche) (STEBICEF), University of Palermo, 90128 Palermo, Italy; gabriella.schiera@unipa.it (G.S.); carlomaria.diliegro@unipa.it (C.M.D.L.); 2 Department of Biomedicine, Neurosciences and Advanced Diagnostics (Dipartimento di Biomedicina, Neuroscienze e Diagnostica avanzata) (Bi.N.D.), University of Palermo, 90127 Palermo, Italy

**Keywords:** thyroid hormones, nuclear and membrane TH receptors, brain development, brain cancer, TH transporters, TH carriers, deiodinases

## Abstract

**Simple Summary:**

Development and function of the mammalian brain clearly require precise regulation of gene expression at both the transcriptional and post-transcriptional level. Thyroid hormones have been recognized to play a fundamental role in these processes, by acting at multiple levels and in different brain cell types, through direct effects on transcription, mediated by nuclear receptors, and also by triggering transduction pathways at the plasma membrane. At the same time, due to their effects on proliferation, differentiation, and cell metabolism, thyroid hormones may have a critical role in different kinds of cancer, including brain cancer.

**Abstract:**

The development and maturation of the mammalian brain are regulated by thyroid hormones (THs). Both hypothyroidism and hyperthyroidism cause serious anomalies in the organization and function of the nervous system. Most importantly, brain development is sensitive to TH supply well before the onset of the fetal thyroid function, and thus depends on the trans-placental transfer of maternal THs during pregnancy. Although the mechanism of action of THs mainly involves direct regulation of gene expression (genomic effects), mediated by nuclear receptors (THRs), it is now clear that THs can elicit cell responses also by binding to plasma membrane sites (non-genomic effects). Genomic and non-genomic effects of THs cooperate in modeling chromatin organization and function, thus controlling proliferation, maturation, and metabolism of the nervous system. However, the complex interplay of THs with their targets has also been suggested to impact cancer proliferation as well as metastatic processes. Herein, after discussing the general mechanisms of action of THs and their physiological effects on the nervous system, we will summarize a collection of data showing that thyroid hormone levels might influence cancer proliferation and invasion.

## 1. Introduction

It is well known since the end of the 19th century that impaired thyroid function can cause mental retardation and other adult neurological disorders [1,2]. 

Consequently, the use of sheep thyroid extracts was introduced in 1891 by Murray [2,3] to treat the hypothyroid condition that had been called myxoedema [4]. About two decades later, 3,5,3′,5′-tetraiodo-L-thyronine (T4 or thyroxine) was isolated from thyroid extracts by Kendall [1,5]. 

Interestingly, it was later found that treatment of pregnant rats with thiouracil (which inhibits the thyroid peroxidase, TPO) delayed ossification in fetuses, thus suggesting that thyroid hormones (THs) from the mother were necessary to drive thyroid-dependent events before the onset of the fetal gland activity [6]. This idea was then confirmed by many groups who demonstrated trans-placental transfer of both L-thyroxine (T4), and L-triiodothyronine (T3), and exposition of fetuses to biologically relevant amounts of free THs during the first trimester of pregnancy (i.e., before the onset of fetal thyroid function) [7,8,9,10,11,12,13]. Notably, it was also demonstrated that maternal thyroxinemia could influence histogenesis of the fetal brain cortex [10]. Moreover, it was found that deiodinases, selenium-containing enzymes involved in TH metabolism (see below), regulate the levels of active thyroid hormones that are transferred, ensuring TH supply to the fetuses while protecting them from an excess of placental transfer (‘barrier effect’) [11,12,13,14,15,16,17,18,19]. 

It has also been clearly established that brain development and maturation, as well as adult neuronal plasticity, strictly depend on dynamic changes in gene expression, and that such modifications in transcriptional programs are in turn determined by chromatin organization [20,21,22,23]. In order to modulate chromatin structure, thus regulating the accessibility of genes to RNA polymerase, a few interrelated mechanisms are required: (i) post-translational modification of histone proteins; (ii) modification of site-specific DNA methylation; (iii) changes in the activity of ATP-dependent chromatin remodeling complexes, such as the chromodomain helicase DNA-binding (Chd) family of enzymes; and (iv) synthesis and incorporation into chromatin of histone variants [21,24,25,26,27,28,29,30]. Now, the nuclear receptors for thyroid hormones (THRs) can bind to chromatin and, depending on the presence of T3 and/or other regulatory factors, can recruit chromatin remodeling complexes and/or histone-modifying activities, thus causing the chromatin structure and gene expression to change [31,32,33,34,35,36,37]. Notably, during their terminal differentiation, cortical neurons acquire a short average spacing of nucleosomes (about 165 bp), while glial cells have a longer nucleosomal length, similar to most other cell types [38,39,40,41]. Although the functional meaning of this property still remains unclear, many years ago it was found that THs are able to induce shortening of the chromatin repeat length in neurons cultured in a chemically defined medium [40]. The short repeat length in differentiated neurons probably depends on the low level of linker H1 histones, even if neuronal chromatin is still able to form fibers [41,42]. 

The effects of THs on the overall nucleosomal spacing suggests that these hormones may influence not only the chromatin organization of single genes but also the general architecture of nuclei. If this is the case, however, alterations of their levels could also be involved in cancer. In cancer cells, chromatin organization is indeed altered in many ways [43,44,45,46], and these modifications can, for example, allow activation of normally silent proto-oncogenes because of loss of an insulated boundary [47].

After summarizing the general mechanisms of action of THs and their physiological effects on the developing and mature nervous system, herein we will discuss a few available data suggesting that, indeed, hypo- and hyperthyroidism might influence brain cancer proliferation and invasion.

## 2. Genomic and Non-Genomic Mechanisms of Action of Thyroid Hormones (THs)

Although the best-known effects of THs are mediated by their nuclear receptors (THRs), it is now clear that TH-dependent signals can start at other sites in the cell, including the plasma membrane. In order to distinguish the slower nuclear responses from other pathways, TH effects are now classified as “genomic” (directly elicited at the chromosomal level) and “non-genomic” (starting at other sites rather than nuclei) [31,37,48,49,50,51,52,53].

### 2.1. Nuclear Receptors for THs (THRs)

**Figure 1:**
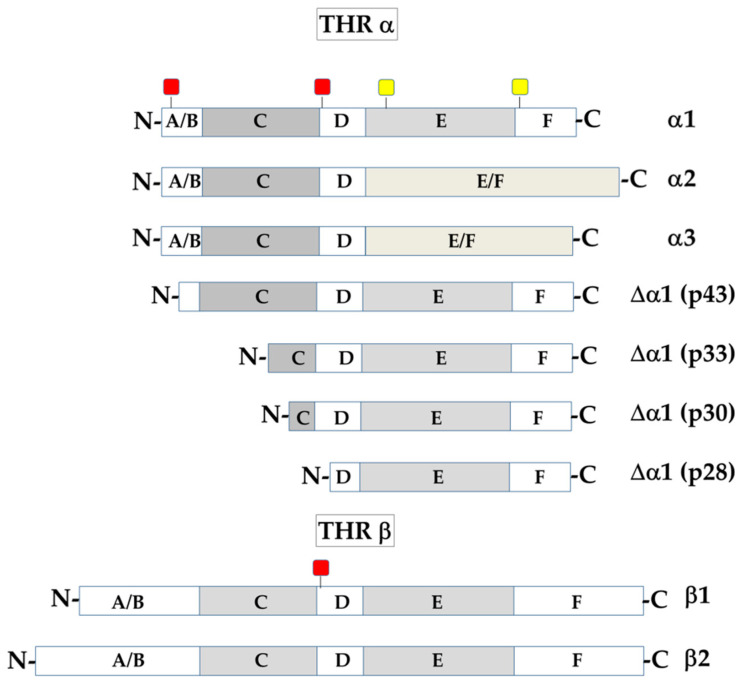
Schematic representation of the domain structure of THRα and THRβ. The full-length TH receptors contain: (i) an N-terminal A/B domain, involved in transcription transactivation; (ii) a DNA-binding domain (C), which recognizes and binds the TH-response elements (TREs), and is also involved in receptor dimerization; (iii) a hinge domain (D) and (iv) an E/F domain that can be involved in binding the hormone, as well as in protein–protein interactions. The full-length THRα1 contains two nuclear localization signals (NLS: red squares) and two atypical mitochondrial import sequences (MIS: yellow squares). The THRα1 shorter forms, which derive from internal AUG translational usage, lack one or both NLS; in these proteins, the localizing effect of the mitochondrial signals is prevalent. THRβ contains only one NLS (red square) in the D domain [66].

THRs are encoded by the c-erbA proto-oncogenes, and belong to a superfamily of nuclear receptors able to recognize short DNA response elements present in the target genes [54,55,56]. Notably, there are two genes (THRA and THRB), present on the human chromosomes 17 and 3, respectively, that are differentially expressed in different tissues of the body (recently reviewed in [37]). The two genes give rise, by alternative splicing, to multiple isoforms of alpha and beta receptors, respectively, that show a similar structure (Figure 1); however, the isoforms known as c-erb-A-α2 and c-erbA-α3 are not able to bind the hormones because the splicing events that generate them eliminate part of the hormone-binding domain [37,57,58,59]. As shown in Figure 1, all the isoforms contain: (i) an N-terminal A/B domain, with modulatory functions, among which a role in transcription transactivation; (ii) a DNA-binding domain (C) that contains two zinc-fingers, each coordinated by a zinc ion, through which it interacts with the DNA double helix at the level of specific motifs, known as TH-response elements (TREs) [13,49,53,60,61]; this region is also involved in receptor dimerization; and (iii) a hinge domain (D) and (iv) an E/F domain, which can be involved in binding the hormone, and in stabilizing THR homo- and hetero-dimers as well as interactions with other proteins [33,37]. A nuclear localization signal (NLS) is also present in the A/B domain of THRα; interestingly, this sequence undergoes post-translational modifications, such as phosphorylation [62], and acetylation [63]. Recently, THR sumoylation has also been evidenced [64].

In addition to the proteins derived by alternative splicing events, different TRα1 isoforms exist that are generated by differential AUG use during translation; as a consequence of using different start codons, these isoforms differ at the N-terminus, while having a conserved hormone-binding domain. TRα1 proteins with a shorter N-end lack one or both the NLS, thus losing the ability to enter the nucleus. On the other hand, in the shorter TRα1 isoforms, atypical mitochondrial import sequences (MISs) (Figure 1) can be preferentially used and can mediate transport to the mitochondria (see below) [65,66].

Although THRs mainly form, like other members of the erbA superfamily, heterodimers with the retinoic acid X receptor (RXR), they can also bind DNA as monomers, homodimers, and even trimers; interestingly, each of these species shows a preference for specific TREs [37,67,68,69].

As mentioned above, THRs can bind to chromatin and recruit chromatin remodeling complexes and/or histone-modifying activities, thus causing the chromatin structure and gene expression to change [31,32,33,34,35,36,37]. In particular, at the level of the so-called ‘positive’ TREs, THR binding, in the absence of THs, recruits “co-repressors”, such as the nuclear corepressor (NCoR), histone deacetylases (HDAC), and methyl-CpG-binding proteins, thus repressing transcription. At the level of the same regulatory DNA sequences, binding of THs to THRs induce the release of co-repressors and binding of “co-activators”, including the nuclear coactivator 1 (NCoA-1), the cAMP-response element binding protein (CREB)-binding protein (CBP), also known as p300, and the p300/CBP-associated factor (p/CAF). The THR-co-activator complexes then elicit transcription activation [33,37,70,71,72,73,74,75,76,77,78,79,80,81,82]. Among the co-activators, central importance has been attributed to proteins with histone acetyl transferase activity (HAT), which can not only modify nucleosomal histones but also non-histone proteins [70,71,83]. 

THR behavior is the opposite at the level of some other TREs: transcription is stimulated in the absence of THs and repressed in their presence; these motifs are defined as ‘negative’ TREs [84,85]. Negative effects of THRs on some genes can also be due to interference with the binding of other transcription factors; for example, hormone-bound THRs can inhibit, in the pituitary gland, the expression of the gene that encodes the β subunit of the thyroid stimulating hormone (TSH). This effect, which is part of the mechanisms responsible for the negative feedback exerted by THs on the hypothalamus-pituitary-thyroid (HPT) axis, seems to be due to a tethering effect that prevents the promoter binding of pituitary-specific transcription factors, such as Pit-1 and GATA2 [86,87,88]. THRs can indeed interact with other proteins and bind to chromatin in indirect ways. Moreover, they can also bind to DNA sequences other than TREs and even outside the gene promoters [37,67,89].

Finally, as mentioned above, the THR ability to enter the nucleus and to bind to DNA and/or to other chromatin proteins also depends on post-translational modifications [62,63,64].

### 2.2. Plasma Membrane Receptors for THs 

The ability of THs to interact with plasma membrane sites was first hypothesized more than 50 years ago [31,51,90,91,92]. In the following decades, it was then clearly demonstrated that extracellular THs can trigger signal transduction pathways that often involve modification of the intracellular concentration of secondary messengers, such as calcium ions [48,51,90,91,92,93,94,95,96,97]. It was also found that an essential TH-binding site of the plasma membrane was αvβ3 integrin, a member of the protein family that anchors the cells to the extracellular matrix (ECM), thus mediating both tissue organization and regulation of cell migration events [97,98,99]. Although initially identified with the same Arg-Gly-Asp (RGD) amino acid sequence responsible for anchoring cells to the ECM, the TH binding site was then found to be more complex. It accommodates two sub-sites: (i) S1, which binds T3, and (ii) S2, which can bind both T3 and T4 but shows a higher affinity for T4, and, in addition, is much more concentrated than T3 outside the cells. T3 binding to S1 elicits a pathway that involves the phosphoinositide-3-kinase (PI3K), and also plays a role in modulating the ability of THRs to shuttle between the cytoplasm and the nucleus. On the other hand, ligand binding to S2 triggers activation of the MAPK/ERK1/2 pathway, via phospholipase C (PLC) and protein kinase C (PKC) [37,99,100,101,102]. As discussed below, activation of intracellular kinases via the MAPK/ERK1/2 pathways can stimulate cell proliferation and might have a role in different cancer types, including glioma [100,103,104,105,106]. Notably, malignant cells express a higher amount of αvβ3 integrin when compared with normal cells [105].

### 2.3. Other TH-Binding Cell Sites 

In addition to THRs and the plasma membrane αvβ3 integrin, THs also interact with other intracellular sites. As mentioned in the previous paragraph, some shorter TRα1 isoforms are generated by starting translation at internal AUG codons. These isoforms (i.e., p43, p33, p30, and p28; Figure 1, THRα) have a conserved C-terminal TH-binding domain but differ at their N-terminus. In particular, they lack one or both NLS that mediate transport of the full-length protein to the nucleus. In the absence of NLS, probably for a change in the overall conformation of the proteins, the effect of atypical mitochondrial import sequences (MISs), present in the more distal part of the molecules, can prevail and mediate transport of at least some of these proteins to mitochondria [65,66]. This is the case of p43 and p28, which localize to the mitochondrial matrix and the inner mitochondrial membrane, respectively [65,66]. Notably, p43 can bind canonical TREs, and four TRE-like motifs are also present in the mitochondrial DNA [65]. Moreover, mitochondrial activity and mitochondriogenesis are both highly stimulated by p43 overexpression [65,107,108]. This observation, together with the finding that TRα2 (one of the two THR isoforms unable to bind TH) can bind to the TREs present in the D-loop of mitochondrial DNA, suggests that THs have direct effects on mitochondrial DNA expression, in addition to the effects mediated by modulation of the expression of nuclear genes encoding mitochondrial proteins [37,109].

On the other hand, p30 has been reported to be palmitoylated and anchored to the inner leaflet of the plasma membrane, in association with caveolin-1 [110]. At this location, p30 binds THs and activates a signal transduction pathway that stimulates a pro-proliferative/pro-survival program, by increasing the intracellular concentrations of calcium, nitric oxide (NO), and cyclic guanosine monophosphate (cGMP), thus triggering sequential activation of protein kinase G II (PKGII), tyrosine kinase Src, extracellular signal-regulated kinase (ERK), and Akt signaling [110].

### 2.4. Transport and Metabolism of THs

After their synthesis in the thyroid gland, L-thyroxine (T4) and triiodo-thyronine (T3) are secreted into the circulation and transported to the target cells by TH distributor proteins (THDPs), mainly high-affinity thyroxine-binding globulin (TBG) and transthyretin (TTR) [111,112,113,114]. In addition to the specialized carriers, albumin and low-density lipoproteins (LDLs) can also bind THs, even if with lower affinity [113]. All these proteins control both the amount of freely exchangeable hormones and uniformity of their distribution in the blood [112,113,114].

Circulating THs enter the cells through membrane carriers, the most important of which are: (i) the monocarboxylate transporters (MCTs) 8 and 10; (ii) the L-type amino acid transporters (LATs); and (iii) the organic anion transporters (OATPs) [82,115,116,117,118,119,120]. As discussed in the next section, mutation of these carriers can cause symptoms of hypothyroidism that can be of particular severity in the nervous system [115,116,118,120].

Although the main circulating form of THs is T4, T3 has been considered the active species as it binds to THRs with the highest affinity [121]. A small amount of T3 is directly produced in the thyroid gland; however, most of it is directly obtained in the target tissues, through the action of selenocysteine-containing deiodinating enzymes (DIOs). Three main members of the DIO family are known: (i) DIO1, a plasma membrane enzyme that catalyzes deiodination of both the outer and inner rings of iodothyronines; (ii) DIO2, an endoplasmic reticulum-resident protein that catalyzes deiodination of the outer (phenolic) ring; and (iii) DIO3, a plasma membrane enzyme that catalyzes deiodination of the inner (thyrosine) ring. In some cellular conditions, such as ischemia/hypoxia, this enzyme rapidly localizes to the nuclear envelope, where it inactivates T3 [37,122,123,124,125]. 

DIO2 seems to play a fundamental role in the TH-dependent negative feedback in the hypothalamus. Tanycytes, specialized ependymal cells located close to the neurons of the paraventricular nucleus (PVN) that produce thyrotropin-releasing hormone (TRH), receive T4 and use DIO2 to transform it into T3. The active hormone then reaches PVN and the pituitary gland, where it inhibits the production of TRH and TSH, respectively [122,126]. As discussed below, DIO2 activity has a critical importance in the brain, where it is enriched in astrocytes. These cells receive T4 and deiodinate it to T3. The active hormone is then transferred to neurons through MCT8 carriers. In the neurons, T3 binds to THRs or is deiodinated by DIO3 [37,116,122]. Noteworthy, this latter enzyme is encoded by an imprinted gene, the expression of which changes during brain development and in the different areas of the brain, thus suggesting a fine stage- and region-specific tuning of the brain’s ability to respond to THs [127].

## 3. Thyroid Hormones and Mammalian Brain Development

Development and maturation of the mammalian brain are extremely sensitive, both before and after birth, to thyroid hormones. Since the 1980s it was indeed recognized that hypothyroid as well as hyperthyroid animals show serious anomalies in the anatomy and function of the brain [31,128,129,130,131,132,133]. Moreover, the TH-sensitive phase of the central nervous system development starts before the onset of the fetal thyroid function, and relies, especially at the beginning, on the TH supply from the mother; therefore, any impairment in maternal TH supply during pregnancy causes irreversible brain alterations and mental retardation in humans [132,133,134,135,136,137,138,139,140,141,142]. In addition, TH deficiency in the perinatal period can cause deafness, due to anomalies of both the peripheral and central auditory system [143,144,145,146,147], also accompanied by general alteration of craniofacial development [148]. In rats, the auditory system mainly develops in the first four weeks after birth, and perinatal TH deficiency results in permanent hearing defects [145,149]. It was also found that some structures of the rat auditory system (e.g., the organ of Corti) could resume when treated with T3 but only when the TH deficiency did not last for prolonged periods in the critical phase [145]. Notably, in the rat auditory area of the cerebral cortex, experimental hypothyroidism causes an increase of callosal projecting neurons [150], probably because of an impairment in the elimination of transient axons that normally accompanies development and projection stabilization [150]. 

Dependence on maternal THs in humans is of special importance during the first trimester of pregnancy, since the fetal thyroid gland function reaches significant levels only in the second trimester; in addition, the HPT axis is not fully functional till 1–2 months after birth; thus, actually, around birth, maternal T4 still represents 30–50% of the T4 measured in the cord blood [151]. Notably, T4 is the main TH that crosses the placenta, thus entering the fetal blood and then the blood–brain barrier, thus accessing the fetal brain [132]. 

Maternal thyroid dysfunction can, in turn, depend on different causes, the most important of which is iodine deficiency [152,153,154,155]. For many years, iodine deficiency was considered a problem restricted to specific geographic areas of the planet (for example, the mountains) and to populations with nutritional deficits, and was finally approached by enriching some foods of mass consumption with iodine in order to increase the iodine supply. For example, in 1994, the World Health Organization (WHO) recommended the addition of iodine to salt used for cooking [155,156,157]. Despite this, iodine deficiency still affects a significant proportion of people in industrialized countries [155,158,159,160,161], with alarming consequences on cognitive functions, as revealed by low intelligence quotient (IQ) and attention deficit hyperactivity disorder (ADHD) in children born from hypothyroid mothers [155]. Even more alarming is the TH-disrupting effects of some chemicals present in the fetal and maternal environment because of pollution or because of their normal presence in widely used industrial products [162,163]. These molecules can act at multiple levels: (i) some of them (for example, perchlorate, nitrate, and thiocyanate) act as inhibitors of the sodium-iodide symporter (NIS), present in the basal membrane of the thyroid follicular cells, and fundamental for iodide uptake into these cells and, as a consequence, for TH synthesis; (ii) other compounds (for example, some pesticides like mancozeb and metiram) inhibit the thyroid peroxidase (TPO), responsible for iodination and synthesis of thyronines [163]; (iii) still other compounds (for example, 4,4′-isopropylidenediphenol, BPA, found in plastic products, such as water bottles and other food containers) can interact with a variety of nuclear hormone receptors. Among the latter, THRs can also be bound, thus activating TH signaling in the absence of T3 [163,164,165,166,167]; and (iv) the chemical similarity among some of these molecules and THs may also cause displacement of hormones from the interacting proteins in the circulation or from the carriers responsible for their entry into the cells. In both cases, an impairment of TH distribution and effects will arise [163]. Finally, it has been underlined that some of these effects might depend on mixtures of chemicals, which do not have any apparent effect when probed as individual molecules but show synergistic effects when present in combination [162,163]. Notably, it has been reported that a mixture of 15 common chemicals, each at concentrations reported in human amniotic fluid, when used as a mixture, could alter thyroid hormone signaling and early brain development in *Xenopus* embryos. In particular, modification of neural proliferation as well as neuron and oligodendrocyte size was noticed [168].

All these observations suggest that, even when their maternal production is normal, T3 and especially T4 must also cross in sufficient amounts a few critical barriers, in order to reach the sensitive cells in the developing brain [169]. As already reported above, T4 is the main TH that crosses the placenta barrier; its transport and delivery to the brain is the next critical step. This happens through the cerebral circulation and hence by delivery across the blood–brain barrier and, in part, through the choroid plexus [132]. Again, the main hormone entering the brain is thus T4, which uses plasma membrane carriers, the most important of which are MCT8 and OATP1C1 [118,120,132,169,170,171]. Among these carriers, MCT8 seems to be specific for TH transport, and indeed mutations in the gene that encodes it (SLC16A2) cause a very rare X-linked disease known as Allan–Herndon–Dudley syndrome (AHDS) or MCT8 deficiency [171]. The affected patients present with moderate to severe intellectual disability and an absence of language, together with hypotonia, bradykinesia, spasticity, and extrapyramidal manifestations [171,172,173]. MCT8 is normally abundant in fetal BBB and its deficiency causes brain hypothyroidism [171]. 

**Figure 2:**
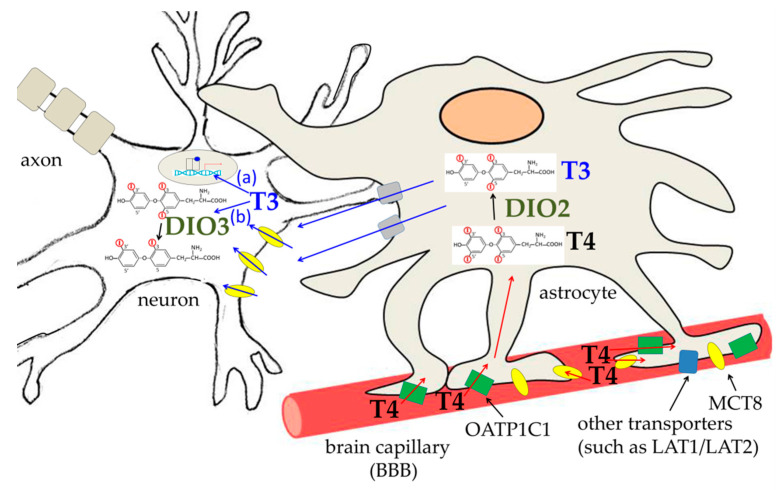
Trafficking of THs to the brain across the blood–brain barrier (BBB). T4 is the main hormone crossing the BBB. It then enters astrocytes through both OATP1C1 (green rectangles) and MCT8 (yellow ovals) transporters, as well as through other transporters (blue rectangles), such as LAT1/LAT2. T3 is much less represented in the blood; a few molecules can perhaps reach astrocytes through MCT8. Once in the astrocytes, T4 is converted to T3 by deiodinase 2 (DIO2). T3 can then exit astrocytes through transporters that are not well characterized (grey rectangles) and enter neurons through MCT8. In the neuron, T3 can enter the nucleus and bind its nuclear receptors (pathway a) or it can be catabolized by DIO3 (pathway b).

Notably, fetal BBB carriers have high preference for T4, being, on the contrary, relatively impermeable to T3 [132]. As mentioned above, indeed, the active hormone T3 is essentially obtained, inside the brain, by astrocytic DIO2 activity: T4 crosses the endothelial cell layer (BBB) and enter astrocytes, which deiodinate it to T3. The active hormone is then transferred to neurons through the crucial MCT8 carriers. In the neurons, T3 will bind to THRs, eliciting its effect on the brain, or will be deiodinated by DIO3 to catabolic products, such as 3,3′-T2 [37,116,122] (Figure 2). Notably, T3 itself stimulates DIO3 synthesis, while inhibiting DIO2 mRNA expression, thus activating a negative feedback loop that opposes an excessive increase of the T3 concentration [127,132,174]. Both in the developing and adult brain, indeed, not only hypothyroidism but also hyperthyroidism can induce severe alterations of brain functions.

During development, all brain cell types are influenced to a various extent by THs, which control a variety of coordinated processes, from neuronal and glial cell proliferation, maturation, and migration to cell survival and programmed cell death. Moreover, THs also regulate neural stem cell fate [175]. A particularly TH-sensitive stage of brain development is the moment at which post-mitotic neurons start migrating to their final destinations, and emitting axonal and dendritic processes, therein establishing and stabilizing their synaptic contacts. A second, and later, important phase is the one in which oligodendrocytes are actively engaged in myelin synthesis. THRs are indeed expressed both in neurons and glial cells, and, although THs can regulate gene expression in brain cells also at the post-transcriptional level [13,132,176,177,178,179,180], most of their effects rely on transcriptional regulation of a variety of genes in both neuronal and glial cells [132,181,182,183]. In agreement with the existence of two main phases of TH action and with the idea that most effects depend on THR-mediated transcriptional effects, it has been found that THR expression, during rat brain development, follows a bimodal pattern, with the first peak at embryonic day 16 (El6) and a later one at postnatal day 6 (P6) [184]. Notably, in both the mammalian and non-mammalian vertebrate brain, TRβ mRNA is expressed later while TRα mRNA is expressed at earlier stages [13,174,185,186,187].

Actually, the main effect of THs seems to be size regulation of the neural progenitor population from which new brain cells derive both in the developing and adult brain [175,188,189]. In agreement with this idea, TH deficiency causes cellular hypoplasia in the rat telencephalon, thus determining, at the end of the brain maturation phase, impairment of complex functions, such as learning and memory capacity [174,190,191]. Similarly, THs promote dopaminergic neuron differentiation from embryonic neural stem cells (NCSs) of the ventral midbrain [192]. On the other hand, hyperthyroidism also inhibits rat neuronal differentiation, thus altering the correct maturation of the fetal cortex [175]. This latter effect is, at least in part, mediated by TH’s effect on the expression of some members of the Hairy and Enhancer of Split (Hes) repressor family. The proteins encoded by these genes contain basic helix-loop-helix (bHLH) domains by which they bind target DNA sequences, then recruiting co-repressors. In doing so, they play a critical role in development, by maintaining progenitor cells and by regulating cell fate decisions [193,194,195]. One of the Hes target genes is Mash1, a basic helix-loop-helix domain-containing regulatory protein able to induce neuronal differentiation [194]. Interestingly, Hes genes have an oscillatory expression in many cell types, including neural stem cells [196,197,198]; for example, Hes1 represses its own expression. Given the very short half-life of both Hes1 mRNA and Hes1 protein, their disappearance soon induces a decrease of the inhibiting effect, and a new round of synthesis, with a periodicity of about 2 h [194,196]. In general terms, Hes genes seem to be required for maintenance but not for generation of NSCs [194]. 

On the other hand, in the early phase of brain development, THs clearly stimulate cell proliferation by upregulating factors, such as Sonic hedgehog (Shh) [131,199]. Shh, in turn, upregulates DIO3 and downregulates DIO2, through DIO2 ubiquitination and degradation [174,200]. In the early phase, THs also induce neuronal differentiation, while, in a later phase, they stimulate differentiation of oligodendroglial precursor cells (OPCs) into myelinating oligodendrocytes, by inducing cell-cycle arrest and transcription of pro-differentiation genes [201,202,203,204]. 

Similarly, during brain development, THs also influence astrocyte differentiation [205,206,207], and astrocytes affect, in turn, neuronal maturation by releasing growth factors and ECM proteins [207].

Interestingly, it has been recently reported that, in a rat model of developmental hypothyroidism, obtained by treating rats from gestation to adulthood with methimazole, autophagy was stimulated in the hippocampal neurons that regulate cognitive functions, with induction of neuroinflammation and impairment of learning and memory capacity This hippocampal neuronal dysfunction could be improved by T4 treatment [208]. 

In order to orchestrate all these processes, THs need to regulate, in a coordinated manner, the expression of complex sets of genes, in different cell types, and in different phases of neurogenesis [175,182,183]. Indeed, hypothyroidism induces alteration in the expression of a variety of genes, among which those encoding the chicken ovalbumin upstream promoter transcription factor 1 (COUP-TF1), involved in the finetuning of T3-stimulated gene expression [175]; neurogranin, a calmodulin-binding synaptic protein involved in learning and memory [209,210]; and many components of the cytoskeleton and ECM [131,211,212,213,214,215], involved in proliferation and migration of nerve cells. It was also found that treatment with anti-thyroid agents, during neuronal development, causes a decrease in the number of parvalbumin-expressing GABAergic interneurons in the mouse cortex and hippocampus [216], as well as a decrease of the total thickness of the somatosensory, auditory, and visual cortices, with a decrease of the methyl-CpG binding protein 2 (MeCP2)-positive neuronal nuclei in the cortical layers II-IV [217]. 

Notably, THs can also affect the overall methylation status of DNA and consequently chromatin structure, by regulating, on the one hand, the expression of DNA methyl-transferases [218,219], and, on the other hand, locus-specific DNA demethylation [220]. Most importantly, it has been suggested that altered levels of THs may affect not only fetal brain development but also brain development of later generations, probably by altering germ line epigenetic information that can then impact on the expression of genes, such as those encoding DNA methyl transferases and deiodinases [221].

As mentioned in the previous sections, THs can also affect cell proliferation, survival, and differentiation through non-genomic mechanisms [222,223]. For example, binding of T4 to integrin αvβ3 activates the MAPK signaling pathway and promotes expansion of progenitors in the embryonic neocortex [131,224]. Notably, it has been reported that tetraiodothyroacetic acid (TETRAC), a deaminated analogue of T4, which inhibits TH binding to integrin αvβ3, completely abolishes the progenitor expansion of the embryonic mouse neocorte induced by integrin αvβ3 activation, thus indicating that expansion requires T4 binding to integrin αvβ3 [224]. Moreover, it has been reported that both T4 and reverse T3 (rT3), but not T3, can regulate the F-actin amount in elongating neurites of cerebellar neurons in culture. This effect can be blocked by peptides able to bind integrins [225].

It is worth noting that THs still have a critical effect on the NSCs of the adult mammalian brain, where these cells are mainly present in the subventricular zone (SVZ) and the subgranular zone (SGZ) of the hippocampus, in a quiescent state (for an extensive and recent review, see [175]). NSCs form a heterogeneous population, with the potential to give rise to both neurons and glial cells, depending on specific signals; for example, SVZ NSCs can generate oligodendroglial precursors (OPCs) after injury [175,204]. This latter effect is mediated by THs [226,227], and probably involves THRβ [227]. Based on this observation, it has been suggested that the use of THRβ agonists might be of help in the treatment of neurodegenerative disorders involving demyelination, such as multiple sclerosis [227]. 

On the other hand, as long as it concerns adult neurogenesis, while there is a general agreement on adult hippocampal neurogenesis (AHN) in the dentate gyrus of rodents, data available for the human brain are somehow discrepant, and the generation of new neurons throughout life still remains to be definitively determined [228,229,230,231,232]. 

## 4. Thyroid Hormones and Brain Cancer

In 1896, in his ‘Original Communication’ on the treatment of inoperable cases of carcinoma of the ‘mamma’ (i.e., breast carcinoma), George Beatson affirmed that it was probably a mistake “to assign to the nervous system the entire regulation of the metabolic changes in the tissues of the body”, and proposed that breast cancer, in particular, might originate from factors produced by the ovaries [233]. This was one of the first suggestions concerning the possible role in cancer of the chemical messengers defined, a decade later, by Ernest Henry Starling as “hormones” [234].

In the following decades a variety of studies focused on what has been called hormonal carcinogenesis [235]. In particular, between the end of the 1970s and the beginning of the 1990s, different studies, performed both in culture and in vivo, suggested that THs had a role in neoplastic transformation, and that hypothyroidism could reverse TH-dependent growth and spread of tumor cells [236,237,238,239,240,241]. For example, a patient affected by a metastatic lung cancer, during chemotherapy, began suffering of cardiac arrhythmia and because of this was also treated with amiodarone HCl. This drug induces thyroid function impairment, and, indeed, some months after starting this therapy, he was hospitalized because of mixedema coma. The patient was treated with thyroxine and recovered. Surprisingly, the tumor mass was reduced and the patient lived for four years after the coma event [239]. Similarly, the experimental induction of a hypothyroid state in mice affected by either spontaneous or xenografted human tumors was often shown to reduce the rate of cancer growth [239]. Moreover, it has been reported that high free T4 (FT4) levels are associated with an increased risk of any solid, lung, and breast cancer [242,243], and that hypothyroidism is associated with an older age of onset of different kinds of cancer (for example, breast and lung cancers) [244,245,246]. In the case of breast cancer, an effect of T3 on cancer cell motility has also been reported [106]. On the other hand, hypothyroidism seems to correlate with an increased risk of colorectal cancer and hepatocellular carcinoma [247]. In particular, THs can control the balance between proliferation and differentiation of colorectal cancer stem cells (CSCs), inducing differentiation and reducing growth, thus acting as an anticancer agent [248,249,250,251].

In Section 2.2, we reported that THs, after binding to αvβ3 integrin, can activate intracellular kinases via the MAPK/ERK1/2 pathways, thus stimulating cell proliferation. This ability might have a role in different cancer types [100,103,104,105,106,245], also because malignant cells express higher amounts of αvβ3 integrin when compared with normal cells [105]. Notably, activation of these pathways affects transcription of a variety of genes encoding proteins involved in different aspects of cancer, such as signal transduction, angiogenesis, regulation of cytoskeleton dynamics, and epithelial–mesenchymal transition [104,105,106]. In addition, T3 binding to the αvβ3 integrin S1 site activates a pathway that involves the phosphoinositide-3-kinase (PI3K), and modulates the ability of THRs to shuttle between the cytoplasm and the nucleus [99,100,101,102]. Moreover, THRs can also directly activate PI3K by binding to its regulatory subunit (p85) [99,251]. It is thus possible to envisage multiple ways through which THs can influence cancer growth and invasion.

However, as discussed below for brain cancers, the molecular mechanisms underlying these effects, and even the consistency of the observations obtained from different experiments have been not completely clarified because the effects of THs might be different in normal and cancer cells, as well as in different kinds of cancer cells [102,252]. 

From this point of view, a relevant topic is the effect of THs on cancer cell response to therapy. It has been reported, for example, that THs improves the effects of chemotherapy in breast cancer patients [253,254]. Similarly, treatment with T3 could enhance the effects of cisplatin and gemcitabine on pancreatic cancer cells [254,255]. However, T3 has opposite effects on hepatocellular carcinoma cells (HCCs) [256] and colon cancer cells, probably by stimulating the expression of P-glycoprotein/multidrug resistance-1 (P-gp/MDR1), a member of the ATP-binding cassette-containing (ABC) transporters that mediate xenobiotic extrusion from cells [257]. These observations are apparently in contrast with the above-mentioned inhibiting effect of THs on the same cancers [247], and suggest that the overall effect of THs can be different in the presence and in the absence of chemotherapy. In any case, the TH effect on P-gp, and consequently on cell efflux of chemotherapeutic drugs, seems to be activated by αvβ3 integrin, since it can be counteracted by the already mentioned TH antagonist TETRAC [257,258], even if THR binding to two closely spaced sequences, present upstream of the transcription start site, has also been reported to be required for the maximal induction of MDR1 gene expression by THs [259]. Notably, activity of P-gp can also be reduced by TETRAC by an αvβ3-mediated reduction of the plasma membrane Na^+^/H^+^ exchanger, and the consequent reduction of intracellular pH [258]. 

Moreover, TETRAC is also able to restore the radiosensitivity of cancer cells, probably by inhibiting TH-induced activation of DNA repair mechanisms in response to the DNA damaging effects of radiation [258]. This TETRAC effect has also been observed in U87MG glioblastoma cells, which, after exposure to the TH antagonist, already show an increased degree of DNA damage in the pre-irradiation state, and reduced capacity of DNA repair in the post-radiation state [260]. Despite these observations in cultured glioblastoma cells, it is not yet clear which effects THs can have on brain cancer response to therapy. On the one hand, indeed, THs have, as discussed above, many different and synergic mechanisms of action, while, on the other hand, brain cancers are highly heterogeneous and complex. Recently, it has become clear that in most solid tumors, a sub-population of cells is present with special properties of therapy resistance. These cells, now called “persisters”, acquire drug tolerance and the ability to resist apoptosis not because of DNA mutations but because of epigenetic processes, such as chromatin remodeling events [261]. Thus THs, due to their ability to modify chromatin organization, can interfere with such events at different levels, and in potentially different ways, also depending on the specific properties of a given persister cell. Thus, depending on the specific kind of tumor and its specific environment, crosstalk might be established between THs and other cancer-expressed factors [262,263,264], thus conditioning the overall effect of THs. 

### 4.1. Effects of THs on Angiogenesis and Brain Cancer Cell Proliferation

Brain cancers are complex and heterogeneous. Most of them derive from glial cells and are called gliomas, then classified as astrocytomas, oligodendrogliomas, ependymomas, and glioastrocytomas depending on their most likely origin, and on the genes expressed in them [265,266,267,268]. 

**Figure 3:**
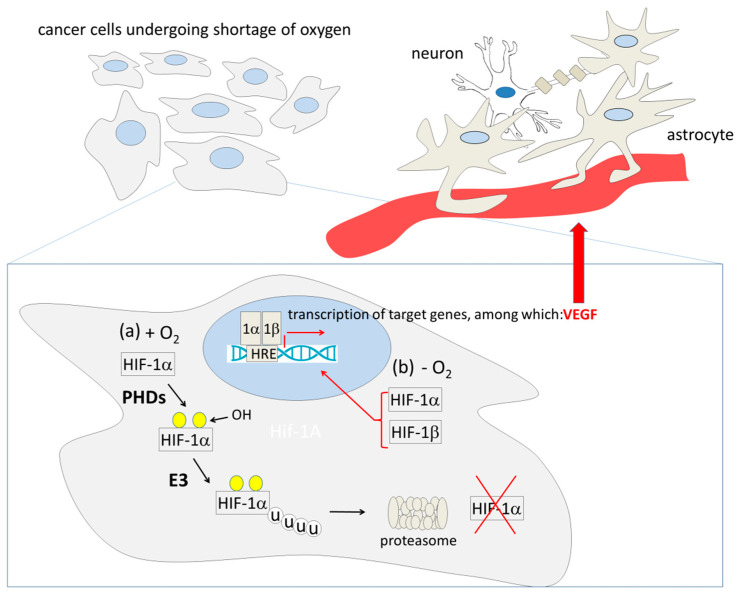
Dependence of angiogenic processes on oxygen shortage and activation of the hypoxia-induced factor 1 (HIF-1). HIF-1 factor is composed by two subunits, HIF-1α and HIF-1β. In normoxic conditions (a, +O_2_), HIF-1α is hydroxylated by prolyl hydroxylase domain proteins (PHDs), poly-ubiquitinated (u) by an E3 ligase, and degraded by the proteasome. When cells (including cancer cells) undergo oxygen shortage (b, -O_2_), PHDs, which use molecular oxygen as a substrate in the hydroxylation reaction, cannot modify any more HIF-1α; as a consequence, HIF-1α is no longer degraded, and combines with HIF-1β; the HIF-1α/HIF-1β dimer enters the nucleus, where it binds to the HIF-1-response elements (HREs) and activates its target genes, including the one encoding vascular endothelial growth factor (VEGF), which in turn stimulates the endothelial cells of the vessels to migrate, proliferate, and differentiate, forming new vessels [273].

The proliferation of glioma cells consumes oxygen, thus generating hypoxia and variable degrees of necrosis [268,269,270]. In turn, oxygen shortage inhibits prolyl hydroxylase domain proteins (PHDs), which use molecular oxygen to hydroxylate their substrates [271], including the α subunit of the hypoxia-inducible transcription factor-1 (HIF-1α). When hydroxylated, HIF-1α is poly-ubiquitinated by the von Hippel–Lindau tumor suppressor protein (pVHL) and degraded by the proteasome [271]. When hydroxylation is inhibited because of hypoxia, HIF-1α is no longer ubiquitinated and degraded; thus, it can heterodimerize with HIF-1β. The dimer enters the nucleus, binds to the hypoxia-response elements (HREs) present in the promoters of hypoxia-regulated genes, and activates them [271,272] (Figure 3). Among the activated genes, some encode angiogenic factors, such as vascular endothelial growth factor (VEGF) and fibroblast growth factor-2 (FGF-2), which stimulate the endothelial cells (ECs) to release proteases that degrade various components of the extracellular matrix, thus allowing ECs themselves to migrate, proliferate, and differentiate, forming new vessels [273]. 

Notably, proliferation of glioma cell lines is stimulated by THs, as demonstrated by accumulation in treated cells of the proliferating cell nuclear antigen (PCNA), and by an increase of radiolabeled thymidine incorporation into newly synthesized DNA in T4-treated cells in culture [274]. This effect is inhibited by TETRAC [274], which, as mentioned above, blocks T4 binding to integrin αvβ3, and inhibits, in the normal developing brain, the expansion of neural progenitors in the neocortex [224]. Similarly, it has been reported that THs have an anti-apoptotic effect in glioma cells [275]. This effect can also be attributed to T4 binding to integrin αvβ3, as it can be counteracted by resveratrol, a compound that induces p53-mediated apoptosis in human cancer cells through interaction with integrin αvβ3 [275]. It is worth noting that, during brain development, THs also have a VEGF/FGF-2-dependent effect on brain angiogenesis [273,276], and treatment of newborn rats for 20 days with propylthiouracil (PTU), an inhibitor of TH synthesis, resulted in decreased complexity and density of brain microvessels [276]. Again, TH’s effects on angiogenesis seem to be mediated by integrin αvβ3, which is also highly expressed in endothelial cells [273].

Moreover, many studies suggest that T4, by binding to integrin αvβ3, stimulates the growth of glioblastoma multiforme (GBM, a grade IV glioma), the most malignant form of glioma [101,275,277]. In agreement with these observations, hypothyroid patients and patients treated with propylthiouracil to inhibit TH synthesis show longer survival than euthyroid ones [100,277]. Similarly, hypothyroidism was recently reported to be associated with favorable survival in patients with brain metastases from other primary cancers [278].

Clinical observations in human astrocytomas have also shown that the expression of THRα1 and/or THRα2 tends to decrease, while the expression of THRβ1 significantly increases, thus suggesting that brain cancers might also be linked to alterations of the combination of nuclear receptors expressed [277,279].

Other studies on glioma cell lines evidenced a more heterogeneous response of cancer cells to THs. It was found, for example, that T3 can induce differentiation in some glioma cell lines, while having a tumor cell type-dependent effect on proliferation, with the more aggressive cells being more sensitive to T3 [280]. 

On the other hand, it has been reported that, in neuroblastoma cells, after binding to its nuclear receptors, T3 blocks RAS-mediated proliferation as well as transcription of genes encoding proteins involved in cell division, such as cyclin D1, thus arresting the cell cycle in G0/G1 [250].

### 4.2. The Possible Role of Deiodinases in Brain Cancer

As mentioned when discussing TH’s effects on brain development, the levels of circulating THs do not necessarily reflect the intracerebral levels of these hormones because of the existence of deiodinases (DIOs), some of which are involved in the production of active T3 from T4, while others catalyze the production of rT3 (from T4) and different forms of T2 (from either T3 or rT3) [37]. Since all these species of THs (including rT3 and T2, once considered inactive species, but instead probably endowed with specific activities) can differently affect cell proliferation and differentiation, by changing the relative concentrations of TH species DIOs can affect the overall response of both normal and cancer cells. Indeed, a variety of studies have demonstrated an involvement of DIOs in carcinogenesis and have suggested, at the same time, that TH effects can be concentration and cell type dependent [281]. In other words, it can be critical to differentiate intratumoral hypo-/hyperthyroidism from general hypo-/hyperthyoridism, as evaluated on the basis of circulating TH levels [281]. In the developing brain, T4 as well as rT3 can modulate neuronal migration and neurite outgrowth by acting on cytoskeletal element polymerization and cell migration ability [225]. Thus, T4 and rT3 might have the same effect on cancer cells, and DIO concentrations and activities, by controlling in turn the concentration of each TH species, which might have a direct impact on carcinogenesis. For example, in a study comparing tissues from different brain cancers (astrocytoma, glioblastoma, and oligodendroglioma), both DIO2 mRNA and activity were reported to be the highest in oligodendroglioma, in comparison with other cancer types, with a positive correlation between the mRNA concentration and enzymatic activity, thus suggesting pretranslational regulation of the DIO2 expression level [281,282,283]. It has also been reported that, on average, the activity of DIO2 is significantly higher in human brain cancer cells with respect to the non-cancer surrounding tissue; however, T3 and T4 concentrations seemed to be significantly lower in gliomas than in non-cancer brain samples [250,284,285]. On the other hand, DIO3 expression is somehow more variable and does not seem to undergo consistent changes in different cancer samples [281].

### 4.3. Cell-to-Cell Communication between Cancer Cells and Microglia

**Figure 4:**
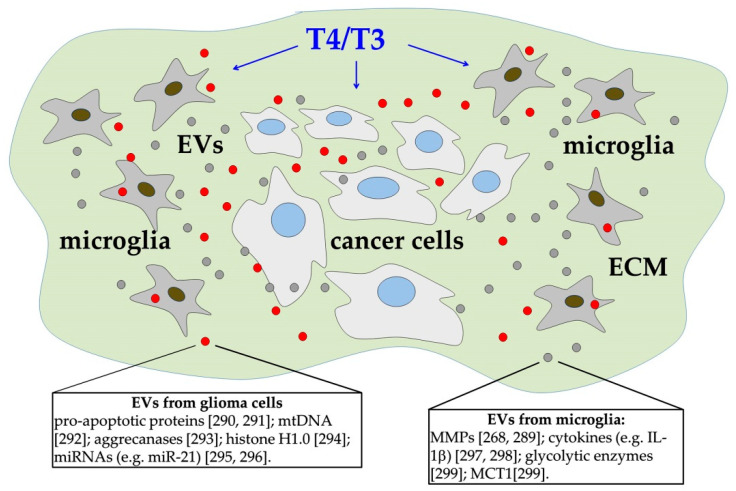
EV-mediated interactions among microglia and cancer cells. Extracellular matrix (ECM) of the glioma tissue is infiltrated by microglial cells, which seem to have a stimulating role in glioma progression. Both microglia and cancer cells produce extracellular vesicles (EVs), which contain different classes of molecules (e.g., proteins and different species of RNA) [268]. A few molecules, contained in EVs released from glioma cells (small red circles), and in EVs released from microglia (small grey circles), are reported in the rectangles, together with some relevant references [268,289,290,291,292,293,294,295,296,297,298,299]. MCT, monocarboxylate transporter; miRNAs, microRNAs; mtDNA, mitochondrial DNA; MMPs, matrix metalloproteinases. It has been suggested that THs (T4/T3) modulate brain cancer growth both directly and indirectly through their effects on microglia [286].

A further interesting point concerns the effects of T3 on microglia, the specialized macrophages of the brain. These latter cells seem, indeed, to be involved in the growth and invasion of gliomas [286] (Figure 4). Resting microglia express only low levels of inflammatory molecules; however, in different pathological conditions, including changes in brain homeostasis, they acquire an ameboid behavior, migrating to the sites of brain damage, and releasing a variety of molecules, such as cytokines, chemokines, and growth factors [287]. Glioma tissue is widely infiltrated by microglia, and these cells clearly have a stimulating role in glioma progression [288]. Actually, the interaction between glioma cells and microglia is a reciprocal one and is probably mostly mediated by extracellular vesicles (EVs) [268] (Figure 4). In particular, it has been found that EVs released from glioma cells can increase cytokine secretion, and the phagocytic capacity of macrophages, as well as an increased production of matrix metalloproteases (MMPs) by microglial cells, thus opening the way for glioma cell migration [268,289]. Notably, THs are important modulators of immune cells, including macrophages, and it has been recently suggested that T3 not only directly modulates brain cancer growth but also indirectly promotes glioma proliferation and migration through its effects on microglia [286].

### 4.4. Aquaporins and Brain Cancer

One of the problems intrinsic to brain cancer derives from its complex relationship with the blood–brain barrier (BBB). On the one hand, indeed, BBB constitutes an obstacle for both identification and treatment of cancer since it opposes drug entering into the brain [300]. On the other hand, however, the presence of brain malignancies causes BBB to become leaky, and induces vasogenic brain edema, which is, indeed, the most serious complication of GBM [301]. Moreover, brain cancer cells are able to trigger both neuronal and glial cell death, associated with cytotoxic edema [301,302]. As discussed in the case of microglia–cancer cell communications, these latter events are probably mediated by release from cancer cells of EVs that contain a collection of different factors, able to stimulate cancer growth, angiogenesis, and invasion, while suppressing immune response [303,304]. Both cytotoxic and vasogenic edemas largely depend on altered expression and/or localization of specific water channels, belonging to the aquaporin family (AQPs). Many AQPs (AQP1, AQP3, AQP4, AQP5, AQP6, AQP8, AQP9, and AQP11) have been identified in the CNS, the most represented of which are AQP1, AQP4, and AQP9 [305]. In particular, AQP4, present both in astrocytes and neurons, has a highly polarized localization in astrocytic endfeet that contact the BBB [304,305,306]. A variety of studies suggested AQP4 involvement in promoting cancer cell migration [307,308,309]. Although the molecular mechanisms underlying AQP4 effects in cancer cells have not been clarified, it has been suggested that AQP4 allows water flow across the plasma membrane at the level of the leading cell protrusions (lamellipodia), with an effect on their number and polarization, thus stimulating cytoskeleton rearrangement and an increase in cell motility [304]. Notably, AQP4 expression was found to be higher in the peritumoral area, which is in the region with the highest ability to invade the surrounding tissue [310]. Recently, it has been reported that AQP4 expression is regulated by THs in astrocytes of the cerebral cortex of newborn and young mice [283]. The same authors also found that T3 treatment significantly downregulates AQP4 in human glioblastoma cells, thus suggesting that higher TH concentration might have a better outcome in reducing AQP4 in brain cancer cells and, hence, in reducing tumor cell migration ability [283].

### 4.5. THs and Cancer Cell Metabolism

One of the properties of cancer cells is reprogramming of energy metabolism, which allows continuous growth, even in hypoxic conditions. The most critical aspect of the metabolic phenotype of cancer cells was described by Warburg almost 70 years ago [311], and consists in increased glycolysis and lactate production, also in the presence of oxygen (aerobic glycolysis) [250,312,313,314]. This property, called the “Warburg effect”, relies, probably, on several metabolic modifications, some of which are still not completely understood. An interesting observation linked, in breast cancer cells, the Warburg effect with TH-dependent induction of one of the pyruvate kinase (PK) isoforms: PKM2 [250,315]. PK catalyzes the last reaction of glycolysis, which is the transfer of a high-energy phosphate group from phosphoenolpyruvate to adenosine diphosphate (ADP), to form adenosine triphosphate (ATP). In mammals, PK is encoded by two genes (PKLR and PKM), each of which gives rise to two PK isoforms [316]. While the PKLR gene only encodes a red blood cell-specific (PKR), and a liver-specific isoform (PKL, also expressed in the kidney), the PKM gene encodes PKM1 and PKM2 isoforms, found in all the other tissues, with PKM2 mainly expressed in proliferating tissues, and especially in cancer cells, where it is involved in the activation of the Warburg effect [316,317,318] but also in gene expression regulation [319,320]. PKM2 expression is induced by the PI3K/mammalian target of rapamycin (mTOR) pathway [316], and thus might be influenced by THs acting at the plasma membrane. Interestingly, recent evidence suggests the existence of a PKM2-mediated link between glucose metabolism and the cell capacity to repair damaged DNA [321]. Surprisingly, PKM2 is phosphorylated, at threonine 328, by the DNA damage-activated ataxia telangiectasia mutated (ATM) kinase, and then promotes DNA repair [321]. Moreover, PKM2 has been reported to be involved in the activation of HIF-1α, critical for metabolic reprogramming of cancer cells [322]. Interestingly, the nuclear functions of PKM2 are regulated by different kinds of non-coding RNAs [320,322]. 

Notably, hypoxia upregulates DIO3 expression, thus increasing the rate of intracellular T3 inactivation, and decreasing oxidative metabolism [323]. In addition, hypoxia induces DIO3 translocation to the nucleus through interaction with the heat shock protein 40 (Hsp40), thus enabling deiodination of T3 at the site of its interaction with the genome [324]. These events are deleterious in cancers, such as ovarian cancer and hepatocellular carcinoma, in which THs act as oncosuppressors [325,326].

Till now, it is not clear whether and at which extent these events are involved in brain cancer; however, PKM2-dependent activation of oncogenic genes has also been reported in the case of glioblastoma, where PKM2 also acts as a histone kinase able to regulate chromatin structure and hence gene expression and tumorigenesis [327]. Given the importance of THs in regulating chromatin organization in brain cells, a role of these hormones in metabolic reprogramming is also highly probable.

## 5. Conclusions and Future Directions

Thyroid hormones have profound effects on many tissues of the body, both during development and in the adult. These effects are mostly triggered by direct regulation of gene expression (genomic effects), mediated by THRs, that can bind to chromatin and, depending on the presence of T3 and/or of a variety of regulatory proteins, can cause chromatin structure and gene expression to change. As discussed above, however, they can also interact with extra-nuclear binding sites, one of which is plasma membrane αvβ3 integrin, thus activating intracellular pathways that can be synergic with THR action but can also be, probably, independent of them. 

During brain development, TH genomic and non-genomic effects show a synergic effect in remodeling chromatin organization and function, thus controlling proliferation, maturation, and metabolism of all the cell types of the nervous system. Moreover, THs seem to also regulate the size of the neural progenitor population from which new brain cells derive both in the developing and adult brain [175,188,189]. Notably, the effects of THs on the brain seem to change in different phases of development. Indeed, in an early phase, they stimulate cell proliferation but also neuronal differentiation, while, in a later phase, they induce cell cycle arrest, and differentiation of myelinating oligodendrocytes, [201,202,203,204]. These stage/time-dependent differences suggest that TH action can be modulated by other regulatory cell factors. 

**Figure 5:**
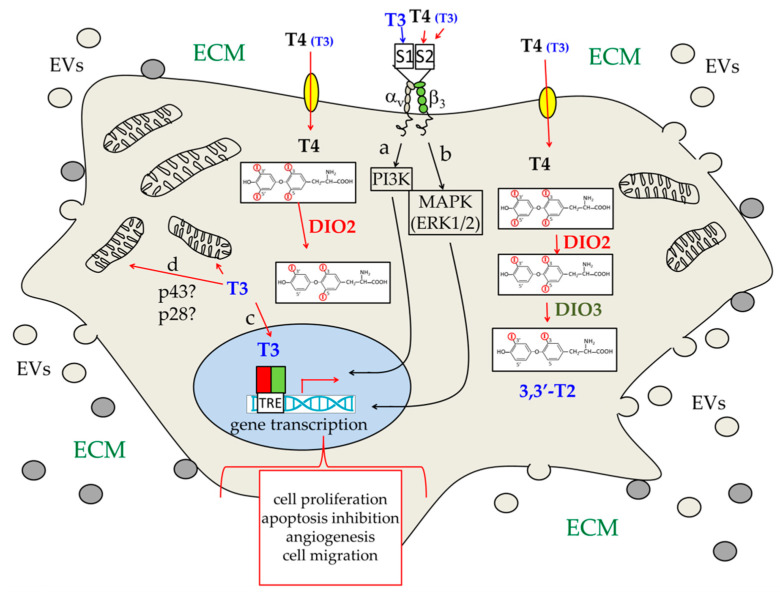
Schematic view of the different hypothetical pathways triggered by THs in a generic brain cancer cell. THs can enter the cells through membrane transporters (yellow ovals), such as MCT8 and OATP1C1. They can also bind to plasma membrane receptors, the best known of which is αvβ3 integrin. T4 is the main form of circulating THs, and thus the main form present in the extracellular matrix (ECM). As a consequence, T4 is also the most abundant TH that enters the cell, and that is able to bind αvβ3 integrin, at the level of the S2 site (red arrow). T3 is much less concentrated outside the cell but can specifically bind to αvβ3 integrin at the level of the S1 site (blue arrow); it can also bind to the S2 site (red arrow), even if it is with much lower affinity than T4. Binding of T3 to the S1 site elicits a signal transduction pathway (a), which involves the phosphoinositide-3-kinas3 (PI3K). On the other hand, binding of T4 (T3) to the S2 site elicits activation of the MAPK/ERK1/2 pathway (b) [99,100,101,102,103,104,105,106]. T4 that enters the cell is converted to T3 by deiodinase 2 (DIO2). T3 can then enter the nucleus (c) and regulate transcription of target genes both directly, by binding to THRs, and indirectly, by interacting with other regulatory proteins [37,67,89]. The a-c regulatory pathways can all influence, directly or indirectly, the transcription of genes involved in different hallmarks of cancer. In addition, T3 has been suggested to be able to directly regulate mitochondrial activities (d), probably by interacting with ∆α1 proteins, such as p43 and p28 [37,65,66,107,108,109]. Finally, as schematically described in Figure 4, brain cancer cells also release extracellular vesicles (EVs; small light brown circles), which can influence other brain cells in the environment, and also receive EVs from the surrounding cells (grey small circles).

Similarly, THs seem to have different effects on different kinds of cancer cells; for example, high free T4 levels are associated with an increased risk of lung and breast cancer [242,243]. Moreover, in the case of breast cancer, T3 stimulates cancer cell motility [106]. Low TH levels, on the contrary, seem to correlate with an increased risk of colorectal cancer and hepatocellular carcinoma [247]. Concerning brain cancer, a TH stimulating effect on different glioma cell lines has been noticed [274]. Moreover, TH effects on angiogenesis have been reported, which seem to be mediated by integrin αvβ3, which is also highly expressed on endothelial cells [273]. For a summary of the different pathways triggered by THs that might have a role in cancer, see Figure 5.

We can conclude that, although many studies suggested a role of THs in cancer growth and metastases, the relationship between general levels of THs and cancer risk is not yet clear-cutting. At the same time, it is possible to hypothesize that the specific TH outcome depends on crosstalk between the hormones and other cancer/environment-expressed factors [262,263,264]. For example, cancer cells express a higher amount of αvβ3 integrin when compared with normal cells [105]. Moreover, as mentioned above, in human astrocytomas, a decrease of THRα1 and/or THRα2, and increase of THRβ1 have been observed, thus suggesting that, at least in the case of these brain cancers, alterations in the combination of the expressed nuclear receptors might also have an importance [277,279].

Actually, as we have discussed, it is probably fundamental to consider that the levels of circulating THs do not necessarily reflect the intra-tissue levels of these hormones because of the existence of so many factors controlling the intracellular levels of THs: (i) TH blood transporters can increase/decrease, thus affecting the actual concentration of free hormones that can enter the cells; (ii) membrane TH carriers can be up/downregulated, thus affecting the entrance of the hormone into the cells, and, as a consequence, also the amount of extracellular hormone that can interact with membrane receptors, and trigger intracellular signal transduction pathways; (iii) different isoforms of deiodinases (DIOs) can be up/downregulated, thus affecting the intracellular TH species concentration; (iv) THR expression and activity can be directly altered or modified because of up/downregulated expression of other critical factors; and (v) all the TH isoforms (including rT3 and T2, once considered inactive species) might differently affect cell proliferation and differentiation. In other words, general hypo-/hyperthyroidism, as evaluated on the basis of circulating TH levels, might give information not in agreement with the real intracellular situation.

Last, but not least, TH effects can be counteracted or, on the contrary, mimicked by individual chemicals or by mixtures of chemicals, present in the environment because of pollution or because of their normal presence in industrial widely used products [162,163]. 

Thus, in summary, THs have a variety of effects on brain cancer cell proliferation, survival, migration, and probably general metabolism, but more experiments are required to better understand the interplay between TH action and environmental/cellular conditions. 

## Data Availability

Not applicable.

## References

[1] Yen P.M. (2001). Physiological and molecular basis of thyroid hormone action. Physiol. Rev..

[2] Pearce J.M.S. (2006). Myxoedema and Sir William Withey Gull (1816–1890). J. Neurol. Neurosurg. Psychiatry.

[3] Murray G.R. (1891). Note on the treatment of myxoedema by hypodermic injections of an extract of the thyroid gland of a sheep. Br. Med. J..

[4] Ord W.M. (1888). Report of a committee of the Clinical Society of London nominated December 14, 1883, to investigate the subject of myxoedema. Trans. Clin. Soc. Lond..

[5] Kendall E.C. (1915). The isolation in crystalline form of the compound containing iodine which occurs in the thyroid: Its chemical nature and physiological activity. Trans. Assoc. Am. Phys..

[6] Weiss R.M., Noback C.R. (1949). The effect of thyroxine and thiouracil on the time of appearance of ossification centers of rat fetuses. Endocrinology.

[7] Gray B., Galton V.A. (1974). The transplacental passage of thyroxine and foetal thyroid function in the rat. Acta Endocrinol..

[8] Man E.B., Serunian S.A. (1976). Thyroid function in human pregnancy. IX. Development or retardation of 7-year-old progeny of hypothyroxinemic women. Am. J. Obstet. Gynecol..

[9] Obregon M.J., Mallol J., Pastor R., de Escobar G.M., del Rey F.E. (1984). Thyroxine and 3,5,5′-triiodothyronine in rat embryos before onset of fetal thyroid function. Endocrinology.

[10] Woods R.J., Sinha A.K., Ekins R. (1984). Uptake and metabolism of thyroid hormones by the rat foetus in early pregnancy. Clin. Sci..

[11] de Escobar G.M., Pastor R., Obregon M.J., del Rey F.E. (1985). Effects of maternal hypothyroidism on the weight and thyroid hormone content of rat embryonic tissues, before and after onset of fetal thyroid function. Endocrinology.

[12] James S.R., Franklin J.A., Kilby M.D. (2007). Placental transport of thyroid hormone. Best Pract. Res. Clin. Endocrinol. Metab..

[13] Di Liegro I. (2008). Thyroid hormones and the central nervous system of mammals (Review). Mol. Med. Rep..

[14] Koopdonk-Kool J.M., De Vijlder J.J., Veenboer G.J., Ris-Stalpers C., Kok J.H., Vulsma T., Boer K., Visser T.J. (1996). Type II and type III deiodinase activity in human placenta as a function of gestation age. J. Clin. Endocrinol. Metab..

[15] Huang S.A., Dorfman D.M., Genest D.R., Salvatore D., Larsen P.R. (2003). Type 3 iodothyronine deiodinase is highly expressed in the human uteroplacental unit and in fetal epithelium. J. Clin. Endocrinol. Metab..

[16] Santini F., Chiovato L., Ghirri P., Lapi P., Mammoli C., Montanelli L., Scartabelli G., Ceccarini G., Coccoli L., Chopra I.J. (1999). Serum iodothyronines in the human fetus and the newborn: Evidence for an important role of placenta in fetal thyroid hormone homeostasis. J. Clin. Endocrinol. Metab..

[17] Chan S., Kachilele S., Hobbs E., Bulmer J.N., Boelaert K., McCabe C.J., Driver P.M., Bradwell A.R., Kester M., Visser T.J. (2003). Placental iodothyronine deiodinase expression in normal and growth-restricted human pregnancies. J. Clin. Endocrinol. Metab..

[18] Moleti M., Trimarchi F., Vermiglio F. (2014). Thyroid physiology in pregnancy. Endocr. Pract..

[19] Hofstee P., Bartho L.A., McKeating D.R., Radenkovic F., McEnroe G., Fisher J.J., Holland O.J., Vanderlelie J.J., Perkins A.V., Cuffe J.S.M. (2019). Maternal selenium deficiency during pregnancy in mice increases thyroid hormone concentrations, alters placental function and reduces fetal growth. J. Physiol..

[20] Gallegos D.A., Chan U., Chen L.F., West A.E. (2018). Chromatin Regulation of Neuronal Maturation and Plasticity. Trends Neurosci..

[21] Goodman J.V., Bonni A. (2019). Regulation of neuronal connectivity in the mammalian brain by chromatin remodeling. Curr. Opin. Neurobiol..

[22] Gray J.M., Spiegel I. (2019). Cell-type-specific programs for activity-regulated gene expression. Curr. Opin. Neurobiol..

[23] Yamada T., Yang Y., Valnegri P., Juric I., Abnousi A., Markwalter K.H., Guthrie A.N., Godec A., Oldenborg A., Hu M. (2019). Sensory experience remodels genome architecture in neural circuit to drive motor learning. Nature.

[24] Marfella C.G.A., Imbalzano A.N. (2007). The Chd Family of Chromatin Remodelers. Mutat. Res..

[25] Strahl B.D., Allis C.D. (2000). The language of covalent histone modifications. Nature.

[26] Speranzini V., Pilotto S., Sixma T.K., Mattevi A. (2016). Touch, act and go: Landing and operating on nucleosomes. EMBO J..

[27] Stillman B. (2018). Histone Modifications: Insights into Their Influence on Gene Expression. Cell.

[28] Roychowdhury T., Chattopadhyay S. (2020). Chemical Decorations of “MARs” Residents in Orchestrating Eukaryotic Gene Regulation. Front. Cell. Dev. Biol..

[29] Talbert P.B., Henikoff S. (2021). Histone variants at a glance. J. Cell Sci..

[30] Goodman J.V., Yamada T., Yang Y., Kong L., Wu D.Y., Zhao G., Gabel H.W., Bonni A. (2020). The chromatin remodeling enzyme Chd4 regulates genome architecture in the mouse brain. Nat. Commun..

[31] Di Liegro I., Savettieri G., Cestelli A. (1987). Cellular mechanism of action of thyroid hormones. Differentiation.

[32] Usala S.J., Young W.S., Morioka H., Nikodem V.M. (1988). The effect of thyroid hormone on the chromatin structure and expression of the malic enzyme gene in hepatocytes. Mol. Endocrinol..

[33] Aranda A., Pascual A. (2001). Nuclear hormone receptors and gene expression. Physiol. Rev..

[34] Lee K.C., Li J., Cole P.A., Wong J., Kraus W.L. (2003). Transcriptional activation by thyroid hormone receptor-beta involves chromatin remodeling, histone acetylation, and synergistic stimulation by p300 and steroid receptor coactivators. Mol. Endocrinol..

[35] Park S.W., Huang W.H., Persaud S.D., Wei L.N. (2009). RIP140 in thyroid hormone-repression and chromatin remodeling of Crabp1 gene during adipocyte differentiation. Nucleic Acids Res..

[36] Gao X., Lee H.Y., Li W., Platt R.J., Barrasa M.I., Ma Q., Elmes R.R., Rosenfeld M.G., Lodish H.F. (2017). Thyroid hormone receptor beta and NCOA4 regulate terminal erythrocyte differentiation. Proc. Natl. Acad. Sci. USA.

[37] Giammanco M., Di Liegro C.M., Schiera G., Di Liegro I. (2020). Genomic and Non-Genomic Mechanisms of Action of Thyroid Hormones and Their Catabolite 3,5-Diiodo-l-Thyronine in Mammals. Int. J. Mol. Sci..

[38] Thomas J.O., Thompson R.J. (1977). Variation in chromatin structure in two cell types from the same tissue: A short DNA repeat length in cerebral cortex neurons. Cell.

[39] Jaeger A.W., Kuenzle C.C. (1982). The chromatin repeat length of brain cortex and cerebellar neurons changes concomitant with terminal differentiation. EMBO J..

[40] Cestelli A., Di Liegro I., Castiglia D., Gristina R., Ferraro D., Salemi G., Savettieri G. (1987). Triiodothyronine-induced shortening of chromatin repeat length in neurons cultured in a chemically defined medium. J. Neurochem..

[41] Clark S.C., Chereji R.V., Lee P.R., Fields R.D., Clark D.J. (2020). Differential nucleosome spacing in neurons and glia. Neurosci. Lett..

[42] Pearson E.C., Butler P.J., Thomas J.O. (1983). Higher-order structure of nucleosome oligomers from short-repeat chromatin. EMBO J..

[43] Dawson M.A., Kouzarides T. (2012). Cancer epigenetics: From mechanism to therapy. Cell.

[44] Audia J.E., Campbell R.M. (2016). Histone Modifications and Cancer. Cold Spring Harb. Perspect. Biol..

[45] Rivera-Reyes A., Hayer K.E., Bassing C.H. (2016). Genomic Alterations of Non-Coding Regions Underlie Human Cancer: Lessons from T-ALL. Trends Mol. Med..

[46] Valencia A.M., Kadoch C. (2019). Chromatin regulatory mechanisms and therapeutic opportunities in cancer. Nat. Cell Biol..

[47] Hnisz D., Weintraub A.S., Day D.S., Valton A.-L., Bak R.O., Li C.H., Goldmann J., Lajoie B.R., Fan Z.P., Sigova A.A. (2016). Activation of proto-oncogenes by disruption of chromosome neighborhoods. Science.

[48] Kavok N.S., Krasilnikova O.A., Babenko N.A. (2001). Thyroxine signal transduction in liver cells involves phospholipase C and phospholipase D activation. Genomic independent action of thyroid hormone. BMC Cell Biol..

[49] Cheng S.Y., Leonard J.L., Davis P.J. (2010). Molecular aspects of thyroid hormone actions. Endocr. Rev..

[50] Vargas-Uricoechea H., Bonelo-Perdomo A., Sierra-Torres C.H. (2014). Effects of thyroid hormones on the heart. Clin. Investig. Arterioscler..

[51] Davis P.J., Goglia F., Leonard J.L. (2016). Nongenomic actions of thyroid hormone. Nat. Rev. Endocrinol..

[52] Taylor E., Heyland A. (2017). Evolution of thyroid hormone signaling in animals: Non-genomic and genomic modes of action. Mol. Cell. Endocrinol..

[53] Flamant F., Cheng S.Y., Hollenberg A.N., Moeller L.C., Samarut J., Wondisford F.E., Yen P.M., Refeto S. (2017). Thyroid Hormone Signaling Pathways: Time for a More Precise Nomenclature. Endocrinology.

[54] Green S., Walter P., Kumar V., Krust A., Bornert J.M., Argos P., Chambon P. (1986). Human oestrogen receptor cDNA: Sequence, expression and homology to v-erb-A. Nature.

[55] Sap J., Muñoz A., Damm K., Goldberg Y., Ghysdael J., Leutz A., Beug H., Vennström B. (1986). The c-erb-A protein is a high-affinity receptor for thyroid hormone. Nature.

[56] Weinberger C., Thompson C.C., Ong E.S., Lebo R., Gruol D.J., Evans R.M. (1986). The c-erb-A gene encodes a thyroid hormone receptor. Nature.

[57] Lazar M.A., Hodin R.A., Darling D.S., Chin W.W. (1988). Identification of a rat c-erbA alpha-related protein which binds deoxyribonucleic acid but does not bind thyroid hormone. Mol. Endocrinol..

[58] Mitsuhashi T., Tennyson G.E., Nikodem V.M. (1988). Alternative splicing generates messages encoding rat c-erbA proteins that do not bind thyroid hormone. Proc. Natl. Acad. Sci. USA.

[59] Lazar M.A. (1993). Thyroid hormone receptors: Multiple forms, multiple possibilities. Endocr. Rev..

[60] Umesono K., Murakami K.K., Thompson C.C., Evans R.M. (1991). Direct repeats as selective response elements for the thyroid hormone, retinoic acid, and vitamin D3 receptors. Cell.

[61] Katz R.W., Subauste J.S., Koenig R.J. (1995). The interplay of half-site sequence and spacing on the activity of direct repeat thyroid hormone response elements. J. Biol. Chem..

[62] Liu Y.Y., Brent G.A. (2018). Posttranslational Modification of Thyroid Hormone Nuclear Receptor by Phosphorylation. Methods Mol. Biol..

[63] Anyetei-Anum C.S., Evans R.M., Back A.M., Roggero V.R., Allison L.A. (2019). Acetylation modulates thyroid hormone receptor intracellular localization and intranuclear mobility. Mol. Cell. Endocrinol..

[64] Liu Y.Y., Brent G.A. (2018). Posttranslational Modification of Thyroid Hormone Nuclear Receptor by Sumoylation. Methods Mol. Biol..

[65] Wrutniak-Cabello C., Casas F., Cabello G. (2017). Mitochondrial T3 receptor and targets. Mol. Cell. Endocrinol..

[66] Anyetei-Anum C.S., Roggero V.R., Allison L.A. (2018). Thyroid hormone receptor localization in target tissues. J. Endocrinol..

[67] Velasco L.F., Togashi M., Walfish P.G., Pessanha R.P., Moura F.N., Barra G.B., Nguyen P., Rebong R., Yuan C., Simeoni L.A. (2007). Thyroid hormone response element organization dictates the composition of active receptor. J. Biol. Chem..

[68] Mengeling B.J., Lee S., Privalsky M.L. (2008). Coactivator recruitment is enhanced by thyroid hormone receptor trimers. Mol. Cell. Endocrinol..

[69] Ramadoss P., Abraham B.J., Tsai L., Zhou Y., Costa-e-Sousa R.H., Ye F., Bilban M., Zhao K., Hollenberg A.N. (2014). Novel mechanism of positive versus negative regulation by thyroid hormone receptor β1 (TRβ1) identified by genome-wide profiling of binding sites in mouse liver. J. Biol. Chem..

[70] Ogryzko V.V., Schiltz R.L., Russanova V., Howard B.H., Nakatani Y. (1996). The transcriptional coactivators p300 and CBP are histone acetyltransferases. Cell.

[71] Spencer T.E., Jenster G., Burcin M.M., Allis C.D., Zhou J., Mizzen C.A., McKenna N.J., Onate S.A., Tsai S.Y., Tsai M.J. (1997). Steroid receptor coactivator-1 is a histone acetyltransferase. Nature.

[72] Jepsen K., Hermanson O., Onami T.M., Gleiberman A.S., Lunyak V., McEvilly R.J., Kurokawa R., Kumar V., Liu F., Seto E. (2000). Combinatorial roles of the nuclear receptor corepressor in transcription and development. Cell.

[73] Cheng S.-Y. (2000). Multiple mechanisms for regulation of the transcriptional activity of thyroid hormone receptors. Rev. Endocr. Metab. Disord..

[74] Wu Y., Koenig R.J. (2000). Gene regulation by thyroid hormones. Trends Endocrinol. Metab..

[75] McKenna N.J., O’Malley B.W. (2002). Combinatorial control of gene expression by nuclear receptors and coregulators. Cell.

[76] Spiegelman B.M., Heinrich R. (2004). Biological control through regulated transcriptional coactivators. Cell.

[77] Smith C.L., O’Malley B.W. (2004). Coregulator function: A key to understanding tissue specificity of selective receptor modulators. Endocr. Rev..

[78] Cheng S.-Y. (2005). Isoform-dependent action of thyroid hormone nuclear receptors: Lessons from knockin mutant mice. Steroids.

[79] Rosenfeld M.G., Lunyak V.V., Glass C.K. (2006). Sensors and signals: A coactivator/corepressor/epigenetic code for integrating signal-dependent programs of transcriptional response. Genes Dev..

[80] Astapova I., Hollenberg A.N. (2013). The in vivo role of nuclear receptor corepressors in thyroid hormone action. Biochim. Biophys. Acta BBA—Gen. Subj..

[81] Vella K.R., Ramadoss P., Costa-e-Sousa R.H., Astapova I., Ye F.D., Holtz K.A., Harris J.C., Hollenberg A.N. (2014). Thyroid hormone signaling in vivo requires a balance between coactivators and corepressors. Mol. Cell. Biol..

[82] Mendoza A., Hollenberg A.N. (2017). New insights into thyroid hormone action. Pharmacol. Ther..

[83] Selvi R.B., Kundu T.K. (2009). Reversible acetylation of chromatin: Implication in regulation of gene expression, disease and therapeutics. Biotechnol. J..

[84] Chatterjee V.K., Lee J.K., Rentoumis A., Jameson J.L. (1989). Negative regulation of the thyroid-stimulating hormone alpha gene by thyroid hormone: Receptor interaction adjacent to the TATA box. Proc. Natl. Acad. Sci. USA.

[85] Hollenberg A.N., Monden T., Flynn T.R., Boers M.E., Cohen O., Wondisford F.E. (1995). The human thyrotropin-releasing hormone gene is regulated by thyroid hormone through two distinct classes of negative thyroid hormone response elements. Mol. Endocrinol..

[86] Nakano K., Matsushita A., Sasaki S., Misawa H., Nishiyama K., Kashiwabara Y., Nakamura H. (2004). Thyroid-hormone-dependent negative regulation of thyrotropin beta gene by thyroid hormone receptors: Study with a new experimental system using CV1 cells. Biochem. J..

[87] Weitzel J.M. (2008). To bind or not to bind—How to down-regulate target genes by liganded thyroid hormone receptor?. Thyroid Res..

[88] Kashiwabara Y., Sasaki S., Matsushita A., Nagayama K., Ohba K., Iwaki H., Matsunaga H., Suzuki S., Misawa H., Ishizuka K. (2009). Functions of PIT1 in GATA2-dependent transactivation of the thyrotropin beta promoter. J. Mol. Endocrinol..

[89] Ayers S., Switnicki M.P., Angajala A., Lammel J., Arumanayagam A.S., Webb P. (2014). Genome-wide binding patterns of thyroid hormone receptor beta. PLoS ONE.

[90] Adamson L.F., Ingbar S.H. (1967). Some properties of the stimulatory effect of thyroid hormones on amino acid transport by embryonic chick bone. Endocrinology.

[91] Goldfine I.D., Simons C.G., Smith G.J., Ingbar S.H. (1975). Cycloleucine transport in isolated rat thymocytes: In vitro effects of triiodothyronine and thyroxine. Endocrinology.

[92] Segal J., Gordon A. (1977). The effects of actinomycin D, puromycin, cycloheximide and hydroxyurea on 30,5,3-triiodo-l-thyronine stimulated 2-deoxy-d-glucose uptake in chick embryo heart cells in vitro. Endocrinology.

[93] Davis P.J., Davis F.B., Lawrence W.D. (1989). Thyroid hormone regulation of membrane Ca^2+^-ATPase activity. Endocr. Res..

[94] Segal J. (1990). In vivo effect of 3,5,30-triiodothyronine on calcium uptake in several tissues in the rat: Evidence for a physiological role of calcium as a first messenger for the prompt action of thyroid hormone at the level of the plasma membrane. Endocrinology.

[95] D’Arezzo S., Incerpi S., Davis F.B., Filippo A., Marino M., Farias R.N., Davis P.J. (2004). Rapid nongenomic effects of 3,5,3′-triiodo-l-thyronine on the intracellular pH of l-6 myoblasts are mediated by intracellular calcium mobilization and kinase pathways. Endocrinology.

[96] Shih A., Zhang S., Cao H.J., Tang H.Y., Davis F.B., Davis P.J., Lin H.Y. (2004). Disparate effects of thyroid hormone on actions of epidermal growth factor and transforming growth factor-alpha are mediated by 3′,5′-cyclic adenosine 5′-monophosphate-dependent protein kinase II. Endocrinology.

[97] Davis P.J., Davis F.B., Cody V. (2005). Membrane receptors mediating thyroid hormone action. Trends Endocrinol. Metab..

[98] Bergh J.J., Lin H.Y., Lansing L., Mohamed N.S., Davis F.B., Moura S., Davis J.P. (2005). Integrin α_v_β_3_ contains a cell surface receptor site for thyroid hormone that is linked to activation of MAPK and induction of angiogenesis. Endocrinology.

[99] Cao X., Kambe F., Moeller L.C., Refeto S., Seo H. (2005). Thyroid hormone induces rapid activation of Akt/protein kinase B-mammalian target of rapamycin-p70S6K cascade through phosphatidylinositol 3-kinase in human fibroblasts. Mol. Endocrinol..

[100] Hercbergs A. (2019). Clinical Implications and Impact of Discovery of the Thyroid Hormone Receptor on Integrin αvβ3-A. Review. Front. Endocrinol..

[101] Lin H.Y., Sun M., Tang H.Y., Lin C., Luidens M.K., Mousa S.A., Incerpi S., Drusano G.L., Davis F.B., Davis P.J. (2009). l-Thyroxine vs. 3,5,3′-triiodo-l-thyronine and cell proliferation: Activation of mitogen-activated protein kinase and phosphatidylinositol 3-kinase. Am. J. Physiol. Cell. Physiol..

[102] Liu Y.C., Yeh C.T., Lin K.H. (2019). Molecular Functions of Thyroid Hormone Signaling in Regulation of Cancer Progression and Anti-Apoptosis. Int. J. Mol. Sci..

[103] Cayrol F., Sterle H.A., Flaque M.C.D., Arcos M.L.B., Cremaschi G.A. (2019). Non-genomic actions of thyroid hormones regulate the growth and angiogenesis of T cell lymphomas. Front. Endocrinol..

[104] Davis P.J., Tang H.Y., Hercbergs A., Lin H.Y., Keating K.A., Mousa S.A. (2018). Bioactivity of Thyroid Hormone Analogs at Cancer Cells. Front. Endocrinol..

[105] Davis P.J., Ashur-Fabian O., Incerpi S., Mousa S.A. (2019). Editorial: Non Genomic Actions of Thyroid Hormones in Cancer. Front. Endocrinol..

[106] Uzair I.D., Grand J.C., Flamini M.I., Sanchez A.M. (2019). Molecular Actions of Thyroid Hormone on Breast Cancer Cell Migration and Invasion via Cortactin/N-WASP. Front. Endocrinol..

[107] Wrutniak C., Cassar-Malek I., Marchal S., Rascle A., Heusser S., Keller J.M., Flechon J., Dauça M., Samarut J., Ghysdael J. (1995). A 43-kDa protein related to c-Erb A alpha 1 is located in the mitochondrial matrix of rat liver. J. Biol. Chem..

[108] Casas F., Rochard P., Rodier A., Cassar-Malek I., Marchal-Victorion S., Wiesner R.J., Cabello G., Wrutniak C. (1999). A variant form of the nuclear triiodothyronine receptor c-ErbAalpha1 plays a direct role in regulation of mitochondrial RNA synthesis. Mol. Cell. Biol..

[109] Morrish F., Buroker N.E., Ge M., Ning X.H., Lopez-Guisa J., Hockenbery D., Portman M.A. (2006). Thyroid hormone receptor isoforms localize to cardiac mitochondrial matrix with potential for binding to receptor elements on mtDNA. Mitochondrion.

[110] Kalyanaraman H., Schwappacher R., Joshua J., Zhuang S., Scott B.T., Klos M., Casteel D.E., Frangos J.A., Dillmann W., Boss G.R. (2014). Nongenomic thyroid hormone signaling occurs through a plasma membrane-localized receptor. Sci. Signal..

[111] Richardson S.J., Wijayagunaratne R.C., D’Souza D.G., Darras V.M., Van Herck S.L. (2015). Transport of thyroid hormones via the choroid plexus into the brain: The roles of transthyretin and thyroid hormone transmembrane transporters. Front. Neurosci..

[112] Janssen S.T., Janssen O.E. (2017). Directional thyroid hormone distribution via the blood stream to target sites. Mol. Cell. Endocrinol..

[113] McLean T.R., Rank M.M., Smooker P.M., Richardson S.J. (2017). Evolution of thyroid hormone distributor proteins. Mol. Cell. Endocrinol..

[114] Rabah S.A., Gowan I.L., Pagnin M., Osman N., Richardson S.J. (2019). Thyroid Hormone Distributor Proteins During Development in Vertebrates. Front. Endocrinol..

[115] Dumitrescu A.M., Liao X.H., Best T.B., Brockmann K., Refeto S. (2004). A novel syndrome combining thyroid and neurological abnormalities is associated with mutations in a monocarboxylate transporter gene. Am. J. Hum. Genet..

[116] Heuer H. (2007). The importance of thyroid hormone transporters for brain development and function. Best Pract. Res. Clin. Endocrinol. Metab..

[117] de Souza E.C., Dias G.R., Cardoso R.C., Lima L.P., Fortunato R.S., Visser T.J., Vaisman M., Ferreira A.C., Carvalho D.P. (2015). MCT8 is Downregulated by Short Time Iodine Overload in the Thyroid Gland of Rats. Horm. Metab. Res..

[118] Strømme P., Groeneweg S., de Souza E.C.L., Zevenbergen C., Torgersbråten A., Holmgren A., Gurcan E., Meima M.E., Peeters R.P., Visser W.E. (2018). Mutated Thyroid Hormone Transporter OATP1C1 Associates with Severe Brain Hypometabolism and Juvenile Neurodegeneration. Thyroid.

[119] Braun D., Wirth E.K., Schweizer U. (2010). Thyroid hormone transporters in the brain. Rev. Neurosci..

[120] Groeneweg S., van Geest F.S., Peeters R.P., Heuer H., Visser W.E. (2020). Thyroid Hormone Transporters. Endocr. Rev..

[121] Wejaphikul K., Groeneweg S., Hilhorst-Hofstee Y., Chatterjee V.K., Peeters R.P., Meima M.E., Visser W.E. (2019). Insight Into Molecular Determinants of T3 vs T4 Recognition From Mutations in Thyroid Hormone Receptor α and β. J. Clin. Endocrinol. Metab..

[122] Drigo R.A., Fonseca T.L., Werneck-de-Castro J.P., Bianco A.C. (2013). Role of the type 2 iodothyronine deiodinase (D2) in the control of thyroid hormone signaling. Biochim. Biophys. Acta BBA—Gen. Subj..

[123] Luongo C., Dentice M., Salvatore D. (2019). Deiodinases and their intricate role in thyroid hormone homeostasis. Nat. Rev. Endocrinol..

[124] Bianco A.C., Dumitrescu A., Gereben B., Ribeiro M.O., Fonseca T.L., Fernandes G.W., Bocco B.M.L.C. (2019). Paradigms of Dynamic Control of Thyroid Hormone Signaling. Endocr. Rev..

[125] Steegborn C., Schweizer U. (2020). Structure and Mechanism of Iodothyronine Deiodinases—What We Know, What We Don’t Know, and What Would Be Nice to Know. Exp. Clin. Endocrinol. Diabetes.

[126] Fekete C., Lechan R.M. (2007). Negative feedback regulation of hypophysiotropic thyrotropin-releasing hormone (TRH) synthesizing neurons: Role of neuronal afferents and type 2 deiodinase. Front. Neuroendocrinol..

[127] Hernandez A., Stohn J.P. (2018). The Type 3 Deiodinase: Epigenetic Control of Brain Thyroid Hormone Action and Neurological Function. Int. J. Mol. Sci..

[128] Legrand J., Henneman G. (1986). Thyroid hormone effects on growth and development. Thyroid Hormone Metabolism.

[129] Dussault J.H., Ruel J. (1987). Thyroid hormones and brain development. Annu. Rev. Physiol..

[130] Rovet J.F. (2014). The role of thyroid hormones for brain development and cognitive function. Endocr. Dev..

[131] Stepien B.K., Huttner W.B. (2019). Transport, Metabolism, and Function of Thyroid Hormones in the Developing Mammalian Brain. Front. Endocrinol..

[132] Miranda A., Sousa N. (2018). Maternal hormonal milieu influence on fetal brain development. Brain Behav..

[133] Prezioso G., Giannini C., Chiarelli F. (2018). Effect of Thyroid Hormones on Neurons and Neurodevelopment. Horm. Res. Paediatr..

[134] Pharoah P.O., Buttfield I.H., Hetzel B.S. (1971). Neurological damage to fetus resulting from severe iodine deficiency during pregnancy. Lancet.

[135] DeLong G.R., Stanbury J.B., Fierro-Benitez A. (1985). Neurological signs in congenital iodine deficiency disorders. Dev. Med. Child Neurol..

[136] Stein S.A., Adams P.M., Shanklin D.R., Mihailoff G.A., Palnitkar M.B. (1991). Thyroid hormone control of brain and motor development: Molecular, neuroanatomical, and behavioural studies. Adv. Exp. Med. Biol..

[137] Heyerdahl S., Kase B.F., Lie S.O. (1991). Intellectual development in children with congenital hypothyroidism in relation to recommended thyroxine treatment. J. Pedriatr..

[138] Haddow J.E., Palomaski G.E., Allan W.C., Williams J.R., Knight G.J., Gagnon J., O’Heir C.E., Mitchell M.L., Hermos R.L., Waisbren S.E. (1999). Maternal thyroid deficiency during pregnancy and subsequent neuropsychological development of the child. N. Engl. J. Med..

[139] Pop V.J., Kuijpens J.L., van Baar A.L. (1999). Low maternal free thyroxine concentrations during early pregnancy are associated with impaired psycomotor development in infancy. Clin. Endocrinol..

[140] Batistuzzo A., Ribeiro M.O. (2020). Clinical and subclinical maternal hypothyroidism and their effects on neurodevelopment, behavior and cognition. Arch. Endocrinol. Metab..

[141] Moog N.K., Entringer S., Heim C., Wadhwa P.D., Kathmann N., Buss C. (2017). Influence of maternal thyroid hormones during gestation on fetal brain development. Neuroscience.

[142] Springer D., Jiskra J., Limanova Z., Zima T., Potlukova E. (2017). Thyroid in pregnancy: From physiology to screening. Crit. Rev. Clin. Lab. Sci..

[143] Meyerhoff W.L. (1979). Hypothyroidism and the ear: Electrophysiological, morphological, and chemical considerations. Laryngoscope.

[144] Rovet J., Walker W., Bliss B., Buchanan L., Ehrlich R. (1996). Long-term sequelae of hearing impairment in congenital hypothyroidism. J. Pediatr..

[145] Knipper M., Zinn C., Maier H., Praetorius M., Rohbock K., Köpschall I., Zimmermann U. (2000). Thyroid hormone deficiency before the onset of hearing causes irreversible damage to peripheral and central auditory systems. J. Neurophysiol..

[146] Ng L., Hernandez A., He W., Ren T., Srinivas M., Ma M., Galton V.A., St Germain D.L., Forrest D. (2009). A protective role for type 3 deiodinase, a thyroid hormone-inactivating enzyme, in cochlear development and auditory function. Endocrinology.

[147] Ng L., Kelley M.W., Forrest D. (2013). Making sense with thyroid hormone—The role of T(3) in auditory development. Nat. Rev. Endocrinol..

[148] Leitch V.D., Bassett J.H.D., Williams G.R. (2020). Role of thyroid hormones in craniofacial development. Nat. Rev. Endocrinol..

[149] Goldey E.S., Kehn L.S., Rehnberg G.L., Crofton K.M. (1995). Effects of developmental hypothyroidism on auditory and motor function in the rat. Toxicol. Appl. Pharmacol..

[150] Lucio R.A., García J.V., Cerezo J.R., Pacheco P., Innocenti G.M., Berbel P. (1997). The development of auditory callosal connections in normal and hypothyroid rats. Cereb. Cortex.

[151] Eng L., Lam L. (2020). Thyroid Function During the Fetal and Neonatal Periods. NeoReviews.

[152] de Escobar G.M.M., Obregón M.J.J., del Rey E.F. (2004). Maternal thyroid hormones early in pregnancy and fetal brain development. Best Pract. Res. Clin. Endocrinol. Metab..

[153] Delange F., Wolff P., Gnat D., Dramaix M., Pilchen M., Vertongen F. (2001). Iodine deficiency during infancy and early childhood in Belgium: Does it pose a risk to brain development?. Eur. J. Pediatr..

[154] Pearce E.N., Lazarus J.H., Moreno-Reyes R., Zimmermann M.B. (2016). Consequences of iodine deficiency and excess in pregnant women: An overview of current knowns and unknowns. Am. J. Clin. Nutr..

[155] Velasco I., Bath S.C., Rayman M.P. (2018). Iodine as essential nutrient during the first 1000 days of life. Nutrients.

[156] Zbigniew S. (2017). Role of Iodine in Metabolism. Recent Pat. Endocr. Metab. Immune Drug Discov..

[157] Charlton K., Skeaff S. (2011). Iodine fortification: Why, when, what, how, and who?. Curr. Opin. Clin. Nutr. Metab. Care.

[158] Zimmermann M.B. (2011). Iodine deficiency in industrialized countries. Clin. Endocrinol..

[159] Trumpff C., De Schepper J., Tafforeau J., Van Oyen H., Vanderfaeillie J., Vandevijvere S. (2013). Mild iodine deficiency in pregnancy in Europe and its consequences for cognitive and psychomotor development of children: A review. J. Trace Elem. Med. Biol..

[160] Rayman M.P., Bath S.C. (2015). The new emergence of iodine deficiency in the UK: Consequences for child neurodevelopment. Ann. Clin. Biochem..

[161] Vanderpump M.P. (2017). Epidemiology of iodine deficiency. Epidemiology of iodine deficiency. Minerva Med..

[162] Mughal B.B., Fini J.B., Demeneix B.A. (2018). Thyroid-disrupting chemicals and brain development: An update. Endocr. Connect..

[163] Demeneix B.A. (2019). Evidence for Prenatal Exposure to Thyroid Disruptors and Adverse Effects on Brain Development. Eur. Thyroid J..

[164] Moriyama K., Tagami T., Akamizu T., Usui T., Saijo M., Kanamoto N., Hataya Y., Shimatsu A., Kuzuya H., Nakao K. (2002). Thyroid hormone action is disrupted by bisphenol A as an antagonist. J. Clin. Endocrinol. Metab..

[165] Zhang Y.-F., Ren X.M., Li Y.Y., Yao X.F., Li C.H., Qin Z.F., Guo L.H. (2018). Bisphenol A alternatives bisphenol S and bisphenol F interfere with thyroid hormone signaling pathway in vitro and in vivo. Environ. Pollut..

[166] Kim M.J., Park Y.J. (2019). Bisphenols and Thyroid Hormone. Endocrinol. Metab..

[167] Yuan N., Wang L., Zhang X., Li W. (2020). Bisphenol A and thyroid hormones: Bibliometric analysis of scientific publications. Medicine.

[168] Fini J.-P., Mughal B.B., Le Mével S., Leemans M., Lettmann M., Spirhanzlova P., Affaticati P., Jenett A., Demeneix B.A. (2017). Human amniotic fluid contaminants alter thyroid hormone signalling and early brain development in *Xenopus embryos*. Sci. Rep..

[169] Landers K., Richard K. (2017). Traversing barriers—How thyroid hormones pass placental, blood-brain and blood-cerebrospinal fluid barriers. Mol. Cell. Endocrinol..

[170] Friesema E.C., Ganguly S., Abdalla A., Fox J.E.M., Halestrap A.P., Visser T.J. (2003). Identification of monocarboxylate transporter 8 as a specific thyroid hormone transporter. J. Biol. Chem..

[171] Grijota-Martínez C., Bárez-López S., Gómez-Andrés D., Guadaño-Ferraz A. (2020). MCT8 Deficiency: The Road to Therapies for a Rare Disease. Front. Neurosci..

[172] Masnada S., Groenweg S., Saletti V., Chiapparini L., Castellotti B., Salsano E., Visser W.E., Tonduti D. (2019). Novel mutations in SLC16A2 associated with a less severe phenotype of MCT8 deficiency. Metab. Brain Dis..

[173] Remerand G., Boespflug-Tanguy O., Tonduti D., Touraine R., Rodriguez D., Curie A., Perreton N., Des Portes V., Sarret C. (2019). RMLX/AHDS Study Group. Expanding the phenotypic spectrum of Allan-Herndon-Dudley syndrome in patients with SLC16A2 mutations. Dev. Med. Child Neurol..

[174] Liu Y.-Y., Brent G.A. (2018). Thyroid hormone and the brain: Mechanisms of action in development and role in protection and promotion of recovery after brain injury. Pharmacol. Ther..

[175] Gothié J.D., Vancamp P., Demeneix B., Remaud S. (2020). Thyroid hormone regulation of neural stem cell fate: From development to ageing. Acta Physiol..

[176] Aniello F., Couchie D., Bridoux A.M., Gripois D., Nunez J. (1991). Splicing of juvenile and adult tau mRNA variants is regulated by thyroid hormone. Proc. Natl. Acad. Sci. USA.

[177] Bajpai M., Chaudhury S. (1999). Transcriptional and post-transcriptional regulation of Na^+^, K^+^-ATPase alpha isoforms by thyroid hormone in the developing rat brain. NeuroReport.

[178] Lorenzo P.I., Ménard C., Miller F.D., Bernal J. (2002). Thyroid hormone-dependent regulation of Tα1 α-tubulin during brain development. Mol. Cell. Neurosci..

[179] Cuadrado A., García-Fernández L.F., Imai T., Okano H., Muñoz A. (2002). Regulation of tau RNA maturation by thyroid hormone is mediated by the neural RNA-binding protein musashi-1. Mol. Cell. Neurosci..

[180] Morte B., Gil-Ibáñez P., Bernal J. (2018). Regulation of Gene Expression by Thyroid Hormone in Primary Astrocytes: Factors Influencing the Genomic Response. Endocrinology.

[181] Dong H., Yauk C.L., Rowan-Carroll A., You S.H., Zoeller R.T., Lambert I., Wade M.G. (2009). Identification of thyroid hormone receptor binding sites and target genes using ChIP-on-chip in developing mouse cerebellum. PLoS ONE.

[182] Gil-Ibanez P., Bernal J., Morte B. (2014). Thyroid hormone regulation of gene expression in primary cerebrocortical cells: Role of thyroid hormone receptor subtypes and interactions with retinoic acid and glucocorticoids. PLoS ONE.

[183] Bernal J. (2017). Thyroid hormone regulated genes in cerebral cortex development. J. Endocrinol..

[184] Perez-Castillo A., Bernal J., Ferreiro B., Pans T. (1985). The early ontogenesis of thyroid hormone receptor in the rat fetus. Endocrinology.

[185] Castiglia D., Cestelli A., Di Liegro C., Bonfanti L., Di Liegro I. (1992). Accumulation of different c-erbA transcripts during rat brain development and in cortical neurons cultured in a synthetic medium. Cell. Mol. Neurobiol..

[186] Forrest D., Sjöberg M., Vennström B. (1990). Contrasting developmental and tissue-specific expression of alpha and beta thyroid hormone receptor genes. EMBO J..

[187] Strait K.A., Schwartz H.L., Perez-Castillo A., Oppenheimer J.M. (1990). Relationship of c-erbA content to tissue triiodothyronine nuclear binding capacity and function in developing and adult rats. J. Biol. Chem..

[188] Lemkine G.F., Raj A., Alfama G., Turque N., Hassani Z., Alegria-Prévot O., Samarut J., Levi G., Demeneix B.A. (2005). Adult neural stem cell cycling in vivo requires thyroid hormone and its alpha receptor. FASEB J..

[189] Fanibunda S.E., Desouza L.A., Kapoor R., Vaidya R.A., Vaidya V.A. (2018). Thyroid Hormone Regulation of Adult Neurogenesis. Vitam. Horm..

[190] Mohan V., Sinha R.A., Pathak A., Rastogi L., Kumar P., Pal A., Godbole M.M. (2012). Maternal thyroid hormone deficiency affects the fetal neocorticogenesis by reducing the proliferating pool, rate of neurogenesis and indirect neurogenesis. Exp. Neurol..

[191] Chen C., Zhou Z., Zhong M., Zhang Y., Li M., Zhang L., Qu M., Yang J., Wang Y., Yu Z. (2012). Thyroid hormone promotes neuronal differentiation of embryonic neural stem cells by inhibiting STAT3 signaling through TRα1. Stem Cells Dev..

[192] Chen C., Ma Q., Chen X., Zhong M., Deng P., Zhu G., Zhang Y., Zhang L., Yang Z., Zhang K. (2015). Thyroid Hormone-Otx2 Signaling Is Required for Embryonic Ventral Midbrain Neural Stem Cells Differentiated into Dopamine Neurons. Stem Cells Dev..

[193] Kageyama R., Ohtsuka T., Kobayashi T. (2007). The Hes gene family: Repressors and oscillators that orchestrate embryogenesis. Development.

[194] Kageyama R., Ohtsuka T., Kobayashi T. (2008). Roles of Hes genes in neural development. Dev. Growth Differ..

[195] Harris L., Guillemot F. (2019). HES1, two programs: Promoting the quiescence and proliferation of adult neural stem cells. Genes Dev..

[196] Hirata H., Yoshiura S., Ohtsuka T., Bessho Y., Harada T., Yoshikawa K., Kageyama R. (2002). Oscillatory expression of the bHLH factor Hes1 regulated by a negative feedback loop. Science.

[197] Kageyama R., Shimojo H., Imayoshi I. (2015). Dynamic expression and roles of Hes factors in neural development. Cell Tissue Res..

[198] Ochi S., Imaizumi Y., Shimojo H., Miyachi H., Kageyama R. (2020). Oscillatory expression of Hes1 regulates cell proliferation and neuronal differentiation in the embryonic brain. Development.

[199] Desouza L.A., Sathanoori M., Kapoor R., Rajadhyaksha N., Gonzalez L.E., Kottmann A.H., Tole S., Vaidya V.A. (2011). Thyroid hormone regulates the expression of the sonic hedgehog signaling pathway in the embryonic and adult Mammalian brain. Endocrinology.

[200] Gereben B., Zavacki A.M., Ribich S., Kim B.W., Huang S.A., Simonides W.S., Zeold A., Bianco A.C. (2008). Cellular and molecular basis of deiodinase-regulated thyroid hormone signaling. Endocr. Rev..

[201] Franco P.G., Silvestroff L., Soto E.F., Pasquini J.M. (2008). Thyroid Hormones Promote Differentiation of Oligodendrocyte Progenitor Cells and Improve Remyelination after Cuprizone-Induced Demyelination. Exp. Neurol..

[202] Dugas J.C., Ibrahim A., Barres B.A. (2012). The T3-Induced Gene KLF9 Regulates Oligodendrocyte Differentiation and Myelin Regeneration. Mol. Cell. Neurosci..

[203] Lee J.Y., Petratos S. (2016). Thyroid Hormone Signaling in Oligodendrocytes: From Extracellular Transport to Intracellular Signal. Mol. Neurobiol..

[204] Breton J.M., Long K.L.P., Barraza M.K., Perloff O.S., Kaufer D. (2021). Hormonal Regulation of Oligodendrogenesis II: Implications for Myelin Repair. Biomolecules.

[205] Dezonne R.S., Lima F.R.S., Trentin A.G., Gomes F.C. (2015). Thyroid hormone and astroglia: Endocrine control of the neural environment. J. Neuroendocrinol..

[206] Das M., Ghosh M., Gharami K., Das S. (2018). Thyroid Hormone and Astrocyte Differentiation. Vitam. Horm..

[207] Morita M., Ikeshima-Kataoka H., Kreft M., Vardjan N., Zorec R., Noda M. (2019). Metabolic Plasticity of Astrocytes and Aging of the Brain. Int. J. Mol. Sci..

[208] Mishra J., Vishwakarma J., Malik R., Gupta K., Pandey R., Maurya S.K., Garg A., Shukla M., Chattopadhyay N., Bandyopadhyay S. (2021). Hypothyroidism Induces Interleukin-1-Dependent Autophagy Mechanism as a Key Mediator of Hippocampal Neuronal Apoptosis and Cognitive Decline in Postnatal Rats. Mol. Neurobiol..

[209] Dowling A.L., Zoeller R.T. (2000). Thyroid hormone of maternal origin regulates the expression of RC3/neurogranin mRNA in the fetal rat brain. Mol. Brain Res..

[210] Dong J., Liu W., Wang Y., Xi Q., Chen J. (2010). Hypothyroidism following developmental iodine deficiency reduces hippocampal neurogranin, CaMK II and calmodulin and elevates calcineurin in lactational rats. Int. J. Dev. Neurosci..

[211] Aniello F., Couchie D., Gripois D., Nunez J. (1991). Regulation of five tubulin isotypes by thyroid hormone during brain development. J. Neurochem..

[212] Nunez J., Couchie D., Aniello F., Bridoux A.M. (1991). Regulation by thyroid hormone of microtubule assembly and neuronal differentiation. Neurochem. Res..

[213] Poddar R., Paul S., Chaudhury S., Sarkar P.K. (1996). Regulation of actin and tubulin gene expression by thyroid hormone during rat brain development. Mol. Brain Res..

[214] Trentin A.G., De Aguiar C.B., Garcez R.C., Alvarez-Silva M. (2003). Thyroid hormone modulates the extracellular matrix organization and expression in cerebellar astrocyte: Effects on astrocyte adhesion. Glia.

[215] Hernandez A., Morte B., Belinchón M.M., Ceballos A., Bernal J. (2012). Critical role of types 2 and 3 deiodinases in the negative regulation of gene expression by T_3_; in the mouse cerebral cortex. Endocrinology.

[216] Gilbert M.E., Sui L., Walker M.J., Anderson W., Thomas S., Smoller S.N., Schon J.P., Phani S., Goodman J.H. (2007). Thyroid hormone insufficiency during brain development reduces parvalbumin immunoreactivity and inhibitory function in the hippocampus. Endocrinology.

[217] Uchida K., Hasuoka K., Fuse T., Kobayashi K., Moriya T., Suzuki M., Katayama N., Itoi K. (2021). Thyroid hormone insufficiency alters the expression of psychiatric disorder-related molecules in the hypothyroid mouse brain during the early postnatal period. Sci. Rep..

[218] Leonard J.L., Farwell A.P. (1997). Thyroid hormone-regulated actin polymerization in brain. Thyroid.

[219] Leonard J.L. (2008). Non-genomic actions of thyroid hormone in brain development. Steroids.

[220] Raj S., Kyono Y., Sifuentes C.J., Arellanes-Licea E.D.C., Subramani A., Denver R.J. (2020). Thyroid Hormone Induces DNA Demethylation in Xenopus Tadpole Brain. Endocrinology.

[221] Martinez M.E., Duarte C.W., Stohn J.P., Karaczyn A., Wu Z., DeMambro V.E., Hernandez A. (2020). Thyroid hormone influences brain gene expression programs and behaviors in later generations by altering germ line epigenetic information. Mol. Psychiatry.

[222] Kyono Y., Subramani A., Ramadoss P., Hollenberg A.N., Bonett R.M., Denver R.J. (2016). Liganded thyroid hormone receptors transactivate the DNA methyltransferase 3a gene in mouse neuronal cells. Endocrinology.

[223] Kyono Y., Sachs L.M., Bilesimo P., Wen L., Denver R.J. (2016). Developmental and Thyroid Hormone Regulation of the DNA Methyltransferase 3a Gene in Xenopus Tadpoles. Endocrinology.

[224] Stenzel D., Wilsch-Brauninger M., Wong F.K., Heuer H., Huttner W.B. (2014). Integrin α_v_β_3_ and thyroid hormones promote expansion of progenitors in embryonic neocortex. Development.

[225] Farwell A.P., Dubord-Tomasetti S.A., Pietrzykowski A.Z., Stachelek S.J., Leonard J.L. (2005). Regulation of cerebellar neuronal migration and neurite outgrowth by thyroxine and 3,3′,5′-triiodothyronine. Brain Res. Dev. Brain Res..

[226] Picou F., Fauquier T., Chatonnet F., Flamant F. (2012). A bimodal influence of thyroid hormone on cerebellum oligodendrocyte differentiation. Mol. Endocrinol..

[227] Saponaro F., Sestito S., Runfola M., Rapposelli S., Chiellini G. (2020). Selective Thyroid Hormone Receptor-Beta (TRβ) Agonists: New Perspectives for the Treatment of Metabolic and Neurodegenerative Disorders. Front. Med..

[228] Dennis C.V., Suh L.S., Rodriguez M.L., Kril J.J., Sutherland G.T. (2016). Human adult neurogenesis across the ages: An immunohistochemical study. Neuropathol. Appl. Neurobiol..

[229] Boldrini M., Fulmore C.A., Tartt A.N., Simeon L.R., Pavlova I., Poposka V., Rosoklija G.B., Stankov A., Arango V., Dwork A.J. (2018). Human Hippocampal Neurogenesis Persists throughout Aging. Cell Stem Cell.

[230] Moreno-Jiménez E.P., Flor-García M., Terreros-Roncal J., Rábano A., Cafini F., Pallas-Bazarra N., Ávila J., Llorens-Martín M. (2019). Adult hippocampal neurogenesis is abundant in neurologically healthy subjects and drops sharply in patients with Alzheimer’s disease. Nat. Med..

[231] Hagihara H., Murano T., Ohira K., Miwa M., Nakamura K., Miyakawa T. (2019). Expression of progenitor cell/immature neuron markers does not present definitive evidence for adult neurogenesis. Mol. Brain.

[232] Seki T. (2020). Understanding the Real State of Human Adult Hippocampal Neurogenesis from Studies of Rodents and Non-human Primates. Front. Neurosci..

[233] Beatson G.T. (1896). On the Treatment of Inoperable Cases of Carcinoma of the Mamma: Suggestions for a New Method of Treatment, with Illustrative Cases. Trans. Med.-Chir. Soc. Edinb..

[234] Henriksen J.H. (2005). Ernest Henry Starling (1866–1927): The scientist and the man. J. Med. Biogr..

[235] Henderson B.E., Feigelson H.S. (2000). Hormonal carcinogenesis. Carcinogenesis.

[236] Kumar M.S., Chiang T., Deodhar D.D. (1979). Enhancing effect of thyroxine on tumor growth and metastases in syngeneic mouse tumor systems. Cancer Res..

[237] Mishkin S.Y., Pollack R., Yalovsky M.A., Morris H.P., Mishkin S. (1981). Inhibition of local and metastatic hepatoma growth and prolongation of survival after induction of hypothyroidism. Cancer Res..

[238] Borek C., Guernsey D.L., Ong A., Edelman I.S. (1983). Critical role played by thyroid hormone in induction of neoplastic transformation by chemical carcinogens in tissue culture. Proc. Natl. Acad. Sci. USA.

[239] Hercbergs A., Leith J.T. (1993). Spontaneous remission of metastatic lung cancer following myxedema coma-an apoptosis-related phenomenon?. J. Natl. Cancer Inst..

[240] Fabian I.D., Rosner M., Fabian I., Vishnevskia-Dai V., Zloto O., Maman E.S., Cohen K., Ellis M., Lin H.-Y., Hercbergs A. (2015). Low thyroid hormone levels improve survival in murine model for ocular melanoma. Oncotarget.

[241] Weingarten C., Jenudi Y., Tshuva R.Y., Moskovich D., Alfandari A., Hercbergs A., Davis P.J., Ellis M., Ashur-Fabian O. (2018). The Interplay Between Epithelial-Mesenchymal Transition (EMT) and the Thyroid Hormones-αvβ3 Axis in Ovarian Cancer. Horm. Cancer.

[242] Khan S.R., Chaker L., Ruiter R., Aerts J.G.J.V., Hofman A., Dehghan A., Franco O.F., Stricker B.H.C., Peeters R.P. (2016). Thyroid function and cancer risk: The Rotterdam Study. J. Clin. Endocrinol. Metab..

[243] Kim E.Y., Chang Y., Lee K.H., Yun J.S., Park Y.L., Park C.H., Ahn J., Shin H., Ryu S. (2019). Serum concentration of thyroid hormones in abnormal and euthyroid ranges and breast cancer risk: A cohort study. Int. J. Cancer.

[244] Hercbergs A.H., Ashur-Fabian O., Garfield D. (2010). Thyroid hormones and cancer: Clinical studies of hypothyroidism in oncology. Curr. Opin. Endocrinol. Diabetes Obes..

[245] Ovčariček P.P., Verburg F.A., Hoffmann M., Iakovou I., Mihailovic J., Vrachimis A., Luster M., Giovanella L. (2021). Higher thyroid hormone levels and cancer. Eur. J. Nucl. Med. Mol. Imaging.

[246] Nisman B., Allweis T.M., Carmon E., Kadouri L., Maly B., Maimon O., Meierovich A., Peretz T. (2020). Thyroid Hormones, Silencing Mediator for Retinoid and Thyroid Receptors and Prognosis in Primary Breast Cancer. Anticancer Res..

[247] Trodello C., Higgins S., Ahadiat O., Wysong A. (2017). Hypothyroidism as a risk factor for cancer: A systematic review and implications for future studies. Cancer Sci. Res. Open Access.

[248] Catalano V., Dentice M., Ambrosio R., Luongo C., Carollo R., Benfante A., Todaro M., Stassi G., Salvatore D. (2016). Activated thyroid hormone promotes differentiation and chemotherapeutic sensitization of colorectal cancer stem cells by regulating Wnt and BMP4 signaling. Cancer Res..

[249] Cicatiello A.G., Ambrosio R., Dentice M. (2017). Thyroid hormone promotes differentiation of colon cancer stem cells. Mol. Cell. Endocrinol..

[250] Goemann I.M., Romitti M., Meyer E.L.S., Wajner S.M., Maia A.L. (2017). Role of thyroid hormones in the neoplastic process: An overview. Endocr. Relat. Cancer.

[251] Hiroi Y., Kim H.H., Ying H., Furuya F., Huang Z., Simoncini T., Noma K., Ueki K., Nguyen N.H., Scanlan T.S. (2006). Rapid nongenomic actions of thyroid hormone. Proc. Natl. Acad. Sci. USA.

[252] Martinez M.B., Ruan M., Fitzpatrick L.A. (2000). Altered response to thyroid hormones by prostate and breast cancer cells. Cancer Chemother. Pharmacol..

[253] Huang J., Jin L., Ji G., Xing L., Xu C., Xiong X., Li H., Wu K., Ren G., Kong L. (2013). Implication from thyroid function decreasing during chemotherapy in breast cancer patients: Chemosensitization role of triiodothyronine. BMC Cancer.

[254] Krashin E., Piekiełko-Witkowska A., Ellis M., Ashur-Fabian O. (2019). Thyroid Hormones and Cancer: A Comprehensive Review of Preclinical and Clinical Studies. Front. Endocrinol..

[255] Michienzi S., Bucci B., Falzacappa C.V., Patriarca V., Stigliano A., Panacchia L., Brunetti E., Toscano V., Misiti S. (2007). 3,3′,5-Triiodo-l-thyronine inhibits ductal pancreatic adenocarcinoma proliferation improving the cytotoxic effect of chemotherapy. J. Endocrinol..

[256] Wang T., Xia L., Ma S., Qi X., Li Q., Xia Y., Tang X., Cui D., Wang Z., Chi J. (2016). Hepatocellular carcinoma: Thyroid hormone promotes tumorigenicity through inducing cancer stem-like cell self-renewal. Sci. Rep..

[257] Davis P.J., Incerpi S., Lin H.Y., Tang H.Y., Sudha T., Mousa S.A. (2015). Thyroid hormone and *P*-glycoprotein in tumor cells. BioMed Res. Int..

[258] Davis P.J., Mousa S.A., Lin H.Y. (2021). Nongenomic Actions of Thyroid Hormone: The Integrin Component. Physiol. Rev..

[259] Kurose K., Saeki M., Tohkin M., Hasegawa R. (2008). Thyroid hormone receptor mediates human MDR1 gene expression-Identification of the response region essential for gene expression. Arch. Biochem. Biophys..

[260] Hercbergs A.H., Lin H.Y., Davis F.B., Davis P.J., Leith J.T. (2011). Radiosensitization and production of DNA double-strand breaks in U87MG brain tumor cells induced by tetraiodothyroacetic acid (tetrac). Cell Cycle.

[261] Vallette F.M., Olivier C., Lézot F., Oliver L., Cochonneau D., Lalier L., Cartron P.F., Heymann D. (2019). Dormant, quiescent, tolerant and persister cells: Four synonyms for the same target in cancer. Biochem. Pharmacol..

[262] Meng R., Tang H.-Y., Westfall J., London D., Cao J.H., Mousa S.A., Luidens M., Hercbergs A., Davis F.B., Davis P.J. (2011). Crosstalk between integrin αvβ3 and estrogen receptor-α is involved in thyroid hormone-induced proliferation in human lung carcinoma cells. PLoS ONE.

[263] Frau C., Godart M., Plateroti M. (2017). Thyroid hormone regulation of intestinal epithelial stem cell biology. Mol. Cell. Endocrinol..

[264] Hercbergs A., Mousa S.A., Leinung M., Lin H.Y., Davis P.J. (2018). Thyroid hormone in the clinic and breast cancer. Horm. Cancer..

[265] Fuller G.N., Scheithauer B.W. (2007). The 2007 revised World Health Organization (WHO) classification of tumors of the central nervous system: Newly codified entities. Brain Pathol..

[266] Louis D.N., Ohgaki H., Wiestler O.D., Cavenee W.K., Burger P.C., Jouvet A., Scheithauer B.W., Kleihues P. (2007). The 2007 WHO classification of tumors of the central nervous system. Acta Neuropathol..

[267] Van den Bent M.J. (2010). Interobserver variation of the histopathological diagnosis in clinical trials on glioma: A clinician’s perspective. Acta Neuropathol..

[268] Schiera G., Di Liegro C.M., Di Liegro I. (2017). Molecular Determinants of Malignant Brain Cancers: From Intracellular Alterations to Invasion Mediated by Extracellular Vesicles. Int. J. Mol. Sci..

[269] Fischer U., Radermacher J., Mayer J., Mehraein Y., Meese E. (2008). Tumor hypoxia: Impact on gene amplification in glioblastoma. Int. J. Oncol..

[270] Irshad K., Mohapatra S.K., Srivastava C., Garg H., Mishra S., Dikshit B., Sarkar C., Gupta D., Chandra P.S., Chattopadhyay P. (2015). A combined gene signature of hypoxia and notch pathway in human glioblastoma and its prognostic relevance. PLoS ONE.

[271] Yang M., Su H., Soga T., Kranc K.R., Pollard P.J. (2014). Prolyl hydroxylase domain enzymes: Important regulators of cancer metabolism. Hypoxia.

[272] Kaelin W.G., Ratcliffe P.J. (2008). Oxygen sensing by metazoans: The central role of the HIF hydroxylase pathway. Mol. Cell..

[273] Pinto M., Soares P., Ribatti D. (2011). Thyroid hormone as a regulator of tumor induced angiogenesis. Cancer Lett..

[274] Davis F.B., Tang H.-Y., Shih A., Keating T., Lansing L., Hercbergs A., Fenstermaker R.A., Mousa A., Mousa S.A., Davis P.J. (2006). Acting via a cell surface receptor, thyroid hormone is a growth factor for glioma cells. Cancer Res..

[275] Lin H.-Y., Tang H.-Y., Keating T., Wu Y.-H., Shih A., Hammond D., Sun M., Hercbergs A., Davis F.B., Davis P.J. (2008). Resveratrol is pro-apoptotic and thyroid hormone is anti-apoptotic in glioma cells: Both actions are integrin and ERK mediated. Carcinogenesis.

[276] Zhang L., Cooper-Kuhn C.M., Nannmark U., Blomgren K., Kuhn H.G. (2010). Stimulatory effects of thyroid hormone on brain angiogenesis in vivo and in vitro. J. Cereb. Blood Flow Metab..

[277] Lin H.Y., Chin Y.-T., Yang Y.-C., Lai H.Y., Wang-Peng J., Liu L.F., Tang H.Y., Davis P.J. (2016). Thyroid Hormone, Cancer, and Apoptosis. Compr. Physiol..

[278] Berghoff A.S., Wippel C., Starzer A.M., Ballarini N., Wolpert F., Bergen E., Wolf P., Steindl A., Widhalm G., Gatterbauer B. (2020). Hypothyroidism correlates with favourable survival prognosis in patients with brain metastatic cancer. Eur. J. Cancer.

[279] Hwang S.L., Lin C.L., Lieu A.S., Hwang Y.F., Howng S.L., Hong Y.R., Chang D.S., Lee K.S. (2008). The expression of thyroid hormone receptor isoforms in human astrocytomas. Surg. Neurol..

[280] Liappas A., Mourouzis I., Zisakis A., Economou K., Lea R.W., Pantos C. (2011). Cell-type-dependent thyroid hormone effects on glioma tumor cell lines. J. Thyroid Res..

[281] Piekiełko-Witkowska A., Nauman A. (2011). Iodothyronine deiodinases and cancer. J. Endocrinol. Investig..

[282] Murakami M., Araki O., Morimura T., Hosoi Y., Mizuma M., Yamada M., Kurihara H., Ishiuchi S., Tamura M., Sasaki T. (2000). Expression of type II iodothyronine deiodinase in brain tumors. J. Clin. Endocrinol. Metab..

[283] Costa L.E.S., Clementino-Neto J., Mendes C.B., Franzon N.H., de Oliveira Costa E., Moura-Neto V., Ximenes-da-Silva A. (2019). Evidence of aquaporin 4 regulation by thyroid hormone during mouse brain development and in cultured human glioblastoma multiforme cells. Front. Neurosci..

[284] Nauman P., Bonicki W., Michalik R., Warzecha A., Czernicki Z. (2004). The concentration of thyroid hormones and activities of iodothyronine deiodinases are altered in human brain gliomas. Folia Neuropathol..

[285] Goemann I.M., Merczyk V.R., Romitti M., Wajner S.M., Maia A.L. (2018). Current concepts and challenges to unravel the role of iodothyronine deiodinases in human neoplasias. Endocr. Relat. Cancer..

[286] Perrotta C., De Palma C., Clementi E., Cervia D. (2015). Hormones and immunity in cancer: Are thyroid hormones endocrine players in the microglia/glioma cross-talk?. Front. Cell. Neurosci..

[287] Wolf S.A., Boddeke H.W.G.M., Kettenmann H. (2017). Microglia in Physiology and Disease. Annu. Rev. Physiol..

[288] Ye X.Z., Xu S.L., Xin Y.H., Yu S.C., Ping Y.F., Chen L., Xiao H.L., Wang B., Yi L., Wang Q.L. (2012). Tumor-associated microglia/macrophages enhance the invasion of glioma stem-like cells via TGF-beta1 signaling pathway. J. Immunol..

[289] De Vrij J., Maas S.L., Kwappenberg K.M., Schnoor R., Kleijn A., Dekker L., Luider T.M., de Witte L.D., Litjens M., van Strien M.E. (2015). Glioblastoma-derived extracellular vesicles modify the phenotype of monocytic cells. Int. J. Cancer.

[290] D’Agostino S., Salamone M., Di Liegro I., Vittorelli M.L. (2006). Membrane vesicles shed by oligodendroglioma cells induce neuronal apoptosis. Int. J. Oncol..

[291] Lo Cicero A., Schiera G., Proia P., Saladino P., Savettieri G., Di Liegro C.M., Di Liegro I. (2011). Oligodendroglioma cells shed microvesicles which contain TRAIL as well as molecular chaperones and induce cell death in astrocytes. Int. J. Oncol..

[292] Guescini M., Genedani S., Stocchi V., Agnati L.F. (2010). Astrocytes and Glioblastoma cells release exosomes carrying mtDNA. J. Neural Transm..

[293] Lo Cicero A., Majkowska I., Nagase H., Di Liegro I., Troeberg L. (2012). Microvesicles shed by oligodendroglioma cells and rheumatoid synovial fibroblasts contain aggrecanase activity. Matrix Biol..

[294] Schiera G., Di Liegro C.M., Saladino P., Pitti R., Savettieri G., Proia P., Di Liegro I. (2013). Oligodendroglioma cells synthesize the differentiation-specific linker histone H1° and release it into the extracellular environment through shed vesicles. Int. J. Oncol..

[295] van der Vos K.E., Abels E.R., Zhang X., Lai C., Carrizosa E., Oakley D., Prabhakar S., Mardini O., Crommentuijn M.H., Skog J. (2016). Directly visualized glioblastoma-derived extracellular vesicles transfer RNA to microglia/macrophages in the brain. Neuro Oncol..

[296] Abels E.R., Maas S.L.N., Nieland L., Wei Z., Cheah P.S., Tai E., Kolsteeg C.J., Dusoswa S.A., Ting D.T., Hickman S. (2019). Glioblastoma-Associated Microglia Reprogramming Is Mediated by Functional Transfer of Extracellular miR-21. Cell Rep..

[297] Bianco F., Pravettoni E., Colombo A., Schenk U., Möller T., Matteoli M., Verderio C. (2005). Astrocyte-derived ATP induce vesicle shedding and IL-1 beta release from microglia. J. Immunol..

[298] Bianco F., Perrotta C., Novellino L., Francolini M., Riganti L., Menna E., Saglietti L., Schuchman E.H., Furlan R., Clementi E. (2009). Acid sphingomyelinase activity triggers microparticle release from glial cells. EMBO J..

[299] Frühbeis C., Fröhlich D., Kuo W.P., Krämer-Albers E.M. (2013). Extracellular vesicles as mediators of neuron-glia communication. Front. Cell. Neurosci..

[300] Zhang F., Xu C.L., Liu C.M. (2015). Drug delivery strategies to enhance the permeability of the blood-brain barrier for treatment of glioma. Drug Des. Dev. Ther..

[301] Dubois L.G., Campanati L., Righy C., D’Andrea-Meira I., Spohr T.C., Porto-Carreiro I., Pereira C.M., Balça-Silva J., Kahn S.A., DosSantos M.F. (2014). Gliomas and the vascular fragility of the blood brain barrier. Front. Cell. Neurosci..

[302] Liang D., Bhatta S., Gerzanich V., Simard J.M. (2007). Cytotoxic edema: Mechanisms of pathological cell swelling. Neurosurg. Focus.

[303] Schiera G., Di Liegro C.M., Di Liegro I. (2015). Extracellular membrane vesicles as vehicles for brain cell-to-cell interactions in physiological as well as pathological conditions. BioMed Res. Int..

[304] Maugeri R., Schiera G., Di Liegro C.M., Fricano A., Iacopino D.G., Di Liegro I. (2016). Aquaporins and Brain Tumors. Int. J. Mol. Sci..

[305] Day R.E., Kitchen P., Owen D.S., Bland C., Marshall L., Conner A.C., Bill R.M., Conner M.T. (2014). Human aquaporins: Regulators of transcellular water flow. Biochim. Biophys. Acta BBA—Gen. Subj..

[306] Nielsen S., Nagelhus E.A., Amiry-Moghaddam M., Bourque C., Agre P., Ottersen O.P. (1997). Specialized membrane domains for water transport in glial cells: High-resolution immunogold cytochemistry of aquaporin-4 in rat brain. J. Neurosci..

[307] Saadoun S., Papadopoulos M.C., Watanabe H., Yan D., Manley G.T., Verkman A.S. (2005). Involvement of aquaporin-4 in astroglial cell migration and glial scar formation. J. Cell Sci..

[308] Ding T., Ma Y., Li W., Liu X., Ying G., Fu L., Gu F. (2011). Role of aquaporin-4 in the regulation of migration and invasion of human glioma cells. Int. J. Oncol..

[309] Ding T., Zhou Y., Sun K., Jiang W., Li W., Liu X., Tian C., Li Z., Ying G., Fu L. (2013). Knockdown a water channel protein, aquaporin-4, induced glioblastoma cell apoptosis. PLoS ONE.

[310] Mou K., Chen M., Mao Q., Wang P., Ni R., Xia X., Liu Y. (2010). AQP-4 in peritumoral edematous tissue is correlated with the degree of glioma and with expression of VEGF and HIF-alpha. J. Neuro-Oncol..

[311] Warburg O. (1956). On the origin of cancer cells. Science.

[312] Hsu P.P., Sabatini D.M. (2008). Cancer cell metabolism: Warburg and beyond. Cell.

[313] Hitosugi T., Chen J. (2014). Post-translational modifications and the Warburg effect. Oncogene.

[314] Arundhathi J.R.D., Mathur S.R., Gogia A., Deo S.V.S., Mohapatra P., Prasad C.P. (2021). Metabolic changes in triple negative breast cancer-focus on aerobic glycolysis. Mol Biol Rep..

[315] Suhane S., Ramanujan V.K. (2011). Thyroid hormone differentially modulates Warburg phenotype in breast cancer cells. Biochem. Biophys. Res. Commun..

[316] Li Y.H., Li X.F., Liu J.T., Wang H., Fan L.L., Li J., Sun G.P. (2018). PKM2, a potential target for regulating cancer. Gene.

[317] Yang W., Zheng Y., Xia Y., Ji H., Chen X., Guo F., Lyssiotis C.A., Aldape K., Cantley L.C., Lu Z. (2012). ERK1/2-dependent phosphorylation and nuclear translocation of PKM2 promotes the Warburg effect. Nat. Cell Biol..

[318] Dayton T.L., Jacks T., Heiden M.G.V. (2016). PKM2, cancer metabolism, and the road ahead. EMBO Rep..

[319] Gao X., Wang H., Yang J.J., Liu X., Liu Z.-R. (2012). Pyruvate Kinase M2 Regulates Gene Transcription by Acting as a Protein Kinase. Mol. Cell.

[320] Puckett D.L., Alquraishi M., Chowanadisai W., Bettaieb A. (2021). The Role of PKM2 in Metabolic Reprogramming: Insights into the Regulatory Roles of Non-Coding RNAs. Int. J. Mol. Sci..

[321] Sizemore S.T., Zhang M., Cho J.H., Sizemore G.M., Hurwitz B., Kaur B., Lehman N.L., Ostrowski M.C., Robe P.A., Miao W. (2018). Pyruvate kinase M2 regulates homologous recombination-mediated DNA double-strand break repair. Cell Res..

[322] Zheng F., Chen J., Zhang X., Wang Z., Chen J., Lin X., Huang H., Fu W., Liang J., Wu W. (2021). The HIF-1α antisense long non-coding RNA drives a positive feedback loop of HIF-1α mediated transactivation and glycolysis. Nat. Commun..

[323] Ciavardelli D., Bellomo M., Crescimanno C., Vella V. (2014). Type 3 deiodinase: Role in cancer growth, stemness, and metabolism. Front. Endocrinol..

[324] Huang L., Yu Z., Zhang T., Zhao X., Huang G. (2014). HSP40 interacts with pyruvate kinase M2 and regulates glycolysis and cell proliferation in tumor cells. PLoS ONE.

[325] Kowalik M.A., Puliga E., Cabras L., Sulas P., Petrelli A., Perra A., Ledda-Columbano G.M., Morandi A., Merlin S., Orrù C. (2020). Thyroid hormone inhibits hepatocellular carcinoma progression via induction of differentiation and metabolic reprogramming. J. Hepatol..

[326] Moskovich D., Alfandari A., Finkelshtein Y., Weisz A., Katzav A., Kidron D., Edelstein E., Veroslavski D., Perets R., Arbib N. (2021). DIO3, the thyroid hormone inactivating enzyme, promotes tumorigenesis and metabolic reprogramming in high grade serous ovarian cancer. Cancer Lett..

[327] Verma H., Cholia R.P., Kaur S., Dhiman M., Mantha A.K. (2021). A short review on cross-link between pyruvate kinase (PKM2) and Glioblastoma Multiforme. Metab. Brain Dis..

